# At the limits of a successful body plan – 3D microanatomy, histology and evolution of *Helminthope* (Mollusca: Heterobranchia: Rhodopemorpha), the most worm-like gastropod

**DOI:** 10.1186/1742-9994-10-37

**Published:** 2013-06-28

**Authors:** Bastian Brenzinger, Gerhard Haszprunar, Michael Schrödl

**Affiliations:** 1 SNSB - Bavarian State Collection of Zoology, Münchhausenstr. 21, Munich 81247, Germany; 2 Department Biology II and GeoBio-Center, Ludwig-Maximilians-Universität München, Großhaderner Str. 2, Planegg-Martinsried 82152, Germany

**Keywords:** Meiofauna, Paedomorphosis, Progenesis, 3d Reconstruction, Euthyneura, Opisthobranch, Pulmonate, Morphology, Phylogeny, Histology

## Abstract

**Background:**

Gastropods are among the most diverse animal clades, and have successfully colonized special habitats such as the marine sand interstitial. Specialized meiofaunal snails and slugs are tiny and worm-shaped. They combine regressive features – argued to be due to progenetic tendencies – with convergent adaptations. Microscopic size and concerted convergences make morphological examination non-trivial and hamper phylogenetic reconstructions. The enigmatic turbellarian-like Rhodopemorpha are a small group that has puzzled systematists for over a century. A preliminary molecular framework places the group far closer to the root of Heterobranchia – one of the major gastropod groups – than previously suggested. The poorly known meiofaunal *Helminthope psammobionta* Salvini-Plawen, 1991 from Bermuda is the most worm-shaped free-living gastropod and shows apparently aberrant aspects of anatomy. Its study may give important clues to understand the evolution of rhodopemorphs among basal heterobranchs versus their previously thought origin among ‘higher’ euthyneuran taxa.

**Results:**

We describe the 3D-microanatomy of *H. psammobionta* using three-dimensional digital reconstruction based on serial semithin histological sections. The new dataset expands upon the original description and corrects several aspects. *Helminthope* shows a set of typical adaptations and regressive characters present in other mesopsammic slugs (called ‘meiofaunal syndrome’ herein). The taxonomically important presence of five separate visceral loop ganglia is confirmed, but considerable further detail of the complex nervous system are corrected and revealed. The digestive and reproductive systems are simple and modified to the thread-like morphology of the animal; the anus is far posterior. There is no heart; the kidney resembles a protonephridium. Data on all organ systems are compiled and compared to *Rhodope*.

**Conclusions:**

*Helminthope* is related to *Rhodope* sharing unique apomorphies. We argue that the peculiar kidney, configuration of the visceral loop and simplicity or lack of other organs in Rhodopemorpha are results of progenesis. The posterior shift of the anus in *Helminthope* is interpreted as a peramorphy, i.e. hypertrophy of body length early in ontogeny. Our review of morphological and molecular evidence is consistent with an origin of Rhodopemorpha slugs among shelled ‘lower Heterobranchia’. Previously thought shared ‘diagnostic’ features such as five visceral ganglia are either plesiomorphic or convergent, while euthyneury and a double-rooted cerebral nerve likely evolved independently in Rhodopemorpha and Euthyneura.

## Introduction

Gastropods are considered to be one of the most diverse major animal groups with respect to ecology and morphology and are the most species-rich taxon outside the arthropod subgroups (see [1,2]). Most gastropods are smaller than 5 millimeters (e.g. [3,4]).

Mesopsammic or meiofaunal gastropods commonly mark this lower size limit. They occupy the microscopic interstices between sand grains of marine subtidal habitats worldwide [5]. Life in these minute spaces between sand grains constrains anatomy, and these taxa commonly show convergent morphologies with other meiofaunal organisms (called ‘meiofaunal syndrome’ herein). This involves a modified body plan with reduction or loss of pigmentation and body appendages (tentacles, shell, gill), an elongation of the body towards a worm-like shape, development of strong epidermal ciliation, adhesive abilities, and the repeated evolution of calcareous spicules as a presumed secondary ‘skeleton’ [6-10]. Other characters are the production of comparatively few but large eggs besides means of direct sperm transfer such as spermatophores or stylets, and the formation of additional ‘accessory’ ganglia in the nervous system. The evolution of several characters and the reduction of size were assumed to be driven by paedomorphosis [11].

There are several lineages of usually shell-less meiofaunal gastropods belonging to the Heterobranchia Gray, 1840. The study of heterobranch phylogeny has recently been revitalized by molecular approaches [12-16]. This taxon covers roughly half of gastropod diversity and contains the majority of ‘seaslugs’, besides all lung-breathing land snails and their aquatic relatives [17,18]. Currently there are less than 100 described meiofaunal heterobranchs (e.g. [7,19]). They belong to at least six independent lineages of seaslugs including some rhodopemorphs, aeolidioidean nudibranchs, cephalaspideans, sacoglossans, and most acochlidians (e.g. [20-26]). Diversity can be expected to be much higher and undescribed species can commonly be found in sand samples from poorly studied areas – these being most of the world [27,28].

The Rhodopemorpha Salvini-Plawen, 1991 [29] or Rhodopidae von Ihering, 1876 [30] is a small group of enigmatic, minute turbellarian-like seaslugs showing characters of the ‘meiofaunal syndrome’, such as the possession of subepidermal spicules. The group deviates much from the general gastropod body plan in completely lacking typical external features such as a shell, mantle cavity, a demarcated foot, visceral sac or tentacles, or the typical gastropod radula [31,32]. Owing to this, the taxonomic history of the group has been much matter of debate. The best-known species, *Rhodope veranii* Kölliker, 1847 [33] lives in the littoral of the Mediterranean [32,34,35]. It was originally placed among nudibranch seaslugs, then redescribed as a flatworm [36], and later placed variously among soleoliferan pulmonate slugs, back among doridoidean nudibranchs, or outside ‘higher’ heterobranchs [29,37-41]. In total, there are only five described species of *Rhodope* from littoral and also mesopsammic habitats around the world (see [32,42]), and little is known about their biology. Recent sampling efforts have discovered at least as many additional morphospecies, according to pigmentation patterns (KM Jörger, NG Wilson pers. comm.).

*Helminthope psammobionta* Salvini-Plawen, 1991 currently is the only described member of the genus [29]. It is a meiofaunal species known only from shallow subtidal sand of Bermuda (western Atlantic). This unpigmented slug represents one of the most aberrant free-living gastropods and an extreme case of adaptation to the interstitial. Living specimens are at first glance hardly recognizable as gastropods: individuals are described as between 1 and 2.5 mm long, externally featureless thread-like worms, with a circular cross-section of 60 to 150 μm [29]. *Helminthope* can be distinguished from other interstitial ‘worms’ such as nemerteans by the combination of comparatively slow, sinuous movement (ciliary gliding, but never backwards), the presence of numerous curved subepidermal calcareous spicules, its conspicuous paired statocysts, and (if detectable) the asymmetric right location of body openings, owing to the original gastropod torsion. In the literature, animals resembling *Helminthope* are only recorded from the southeastern United States (as *Rhodope* sp., see [9,43]). However, recent samplings have also retrieved undescribed species from other subtropic or tropic seas (KM Jörger, NG Wilson, pers. comm.; BB, MS - own unpublished data), some of which possess unique cross-shaped spicules and may be a third, still unnamed lineage of Rhodopemorpha (see [9,42]). This indicates that the genus is much more widespread than previously thought.

*Helminthope* was originally placed among Rhodopemorpha [29], which was later doubted on the basis of ultrastructural characters [41]. Preliminary molecular data recover Rhodopemorpha as monophyletic and place the slug taxon as part of the still unresolved but paraphyletic ‘lower Heterobranchia’ or ‘Allogastropoda’. More specifically, Rhodopemorpha is currently indicated as sister to the Murchisonellidae Casey, 1904 [44], a taxon of minute marine snails with high-spired shells that can be retraced from fossils back to the Triassic [45]. This phylogenetic position is far from the previously suggested origins among ‘higher’ heterobranchs, the Euthyneura Spengel, 1881. These comprise members with more or less detorted, i.e. ‘euthyneuran’ nervous systems and were also named Pentaganglionata Haszprunar, 1985 due to their possession of five ganglia on the visceral loop [17], characters that have been given much weight in traditional taxonomy of gastropods. This leads to the observation that rhodopemorphs are, in an anatomical sense, unambiguously ‘euthyneurous’ and ‘pentaganglionate’ (according to the original description of *Helminthope* and data on *Rhodope*, [35]), but not a member of the namesake clades [15].

Due to their small size, examinations of micromolluscs are often limited to SEM study of hard parts like shells or radulae. If these features are lacking as in Rhodopemorpha, histological examination is a useful tool to characterize anatomical features. Computerized three-dimensional reconstruction facilitates understanding of complex anatomical features and can be based on histology (besides other methods), thus including information on the level of tissues and often even cells. Studies based on serial semithin sections have lately provided systematists with reliable and detailed anatomical datasets of complex organs or even entire organisms of minute taxa, improving knowledge of species that often occupy key positions in otherwise proposed phylogenies. In gastropod research, such studies have been published mainly for minute Heterobranchia (e.g. [22,24,46-53]).

In this paper, we explore at a semi-thin histological scale the 3D-visualized microanatomy of *Helminthope psammobionta*, correcting and supplementing the original description ([29], Table 1) and establishing a detailed and comprehensive dataset for comparison to *Rhodope.* This enables us to characterize presently known rhodopemorph genera. We discuss rhodopemorph evolution towards extreme body shape via putative progenetic processes. Finally, we summarize current heterobranch phylogeny and discuss placement of rhodopemorphs and compare anatomy of rhodopemorphs to other heterobranchs, in order to reconstruct and discuss their phylogenetic position and evolution as “lower” versus “higher” heterobranchs.

**Table 1:** **Differences between originally described characters of *Helminthope psammobionta *and results of this study**

	**Salvini-Plawen, 1991 **[29]	**This study**
Optic and buccal ganglia innervated by	branches of ‘terminal cerebropleural connective’*	opg: optic nerve parallel to N4 bg: ventral sides of cpg
Buccal ganglia located	behind statocysts/pedal ganglia	anterior to pedal ganglia
Pedal ganglia	with pronounced anterior lobes	spheroid
Visceral = abdominal ganglion	with ‘chiasma of fibres’ indicating streptoneury	?without traces of streptoneury
Paired visceral nerves	with anterior-running branches [29:Figure 4]	not branching
Postcerebral accessory ganglia	not described	on N4, pedal nerve, ?opgn
Vesicle filled with spermatozoa is	a ‘spermatheca’ distal to nidamental glands	an ampulla proximal to nidamental glands
Gonad	‘appears ramified’; protandric, possibly gonochoric	~ tubular; hermaphroditic (possibly protandric)
Externally visible tube below CNS is	anteriormost part of genital system (genital opening ‘still appears to be absent’)	single tubular salivary gland
Ciliated opening at right body side	is ‘(reduced) mantle cavity’ (= anus and protonephridiopore)	is genital opening
Intestine located	approx. 100 μm behind visceral ganglion	in posterior fifth of body
Ventroterminal adhesive gland	not detected/ missing	present

**Figure 1:**
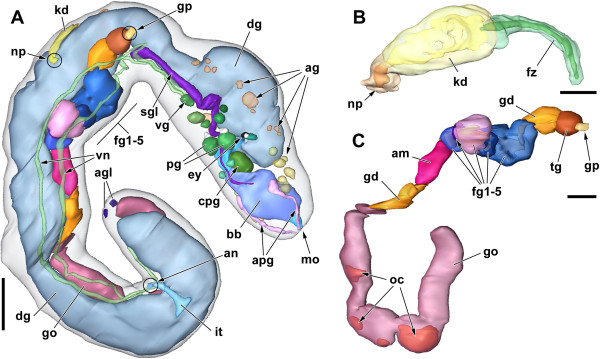
**3D reconstruction of *H. psammobionta *(ZSM Mol-19992019/2) showing organization of major organ systems.** Anterior to the right. **A**: Right view of complete, moderately contracted specimen. **B**: Kidney of same specimen, dorsal view. **C**: Reproductive system. Scale bars: **A**, 100 μm; **B**, 25 μm; **C**, 50 μm. Abbreviations: ag, accessory ganglia; agl, caudal adhesive gland; am, ampulla; an, anus; apg, anterior pedal glands; bb, buccal bulb; cpg, cerebropleural ganglion; dg, digestive gland; ey, eye; fg1-5, female glands (proximal to distal); fz, presumed filter zone; gd, (undifferentiated) gonoduct; go, gonad; gp, genital pore; it, intestine; kd, kidney; mo, mouth opening; np, nephropore; oc, oocytes; pg, pedal ganglia; sgl, salivary gland; tg, ‘terminal’ gland; vg, visceral ganglion; vn, visceral nerves.

Abbreviations as in Figure 1. (*: should be pleuropedal connective?).

## Results

### General morphology and histology

**Figure 2:**
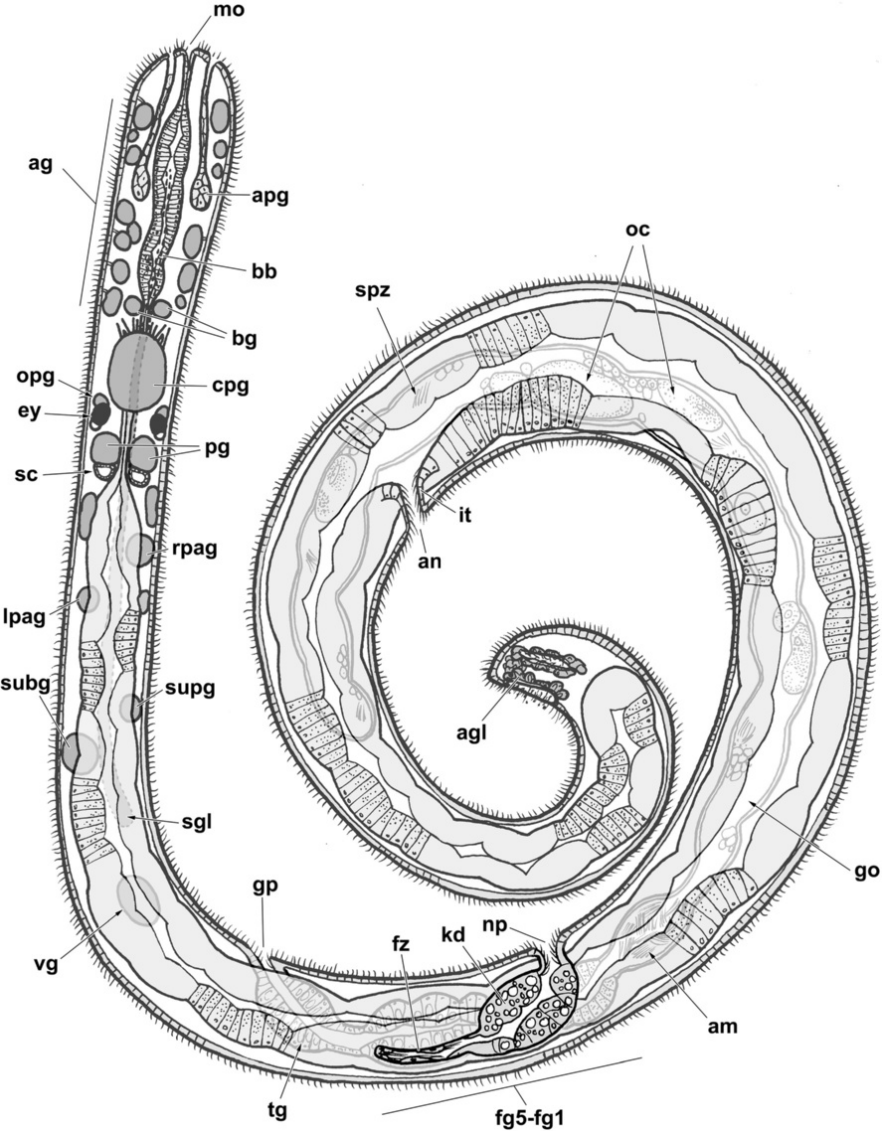
**Schematic dorsal view of *H. psammobionta *(based on specimen shown in Figure **3**A’).** Abbreviations: ag, accessory ganglia; agl, caudal adhesive gland; am, ampulla; an, anus; apg, anterior pedal glands; bb, buccal bulb; bg, buccal ganglia; cpg, cerebropleural ganglion; dg, digestive gland; ey, eye; fg1-5, female glands (proximal to distal); fz, presumed filter zone; go, gonad; gp, genital pore; it, intestine; kd, kidney; lpag, left parietal ganglion; mo, mouth opening; np, nephropore; oc, oocytes; opg, optic ganglion; pg, pedal ganglia; rpag, right parietal ganglion; sc, statocyst; sgl, salivary gland; spz, spermatozoa; subg, subintestinal ganglion; supg, supraintestinal ganglion; tg, ‘terminal’ gland; vg, visceral ganglion.

**Figure 3:**
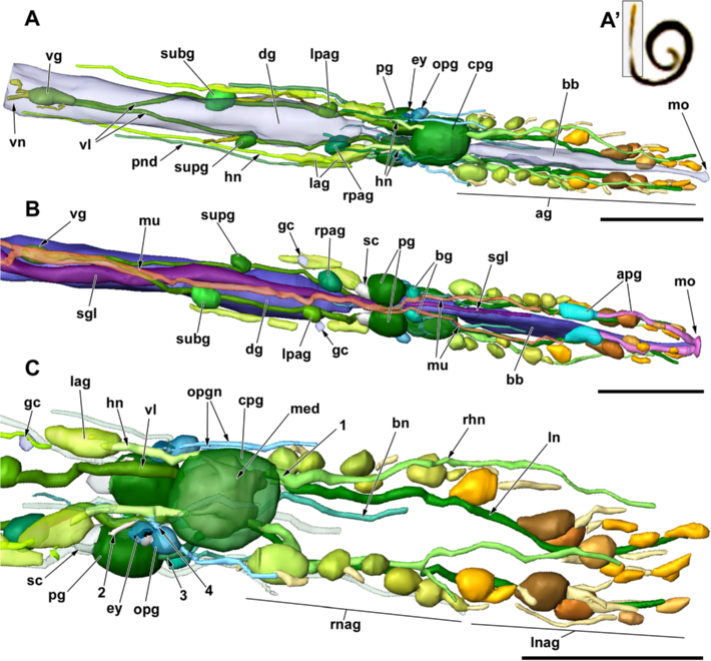
**3D reconstruction of the anterior end of an extended *H. psammobionta *(ZSM Mol-19992020/2) showing details of the central nervous system (cns).** Anterior to the right. **A**: Dorsal view of cns. Digestive system transparent, pedal nerves omitted. **A’**: The reconstructed specimen prior to sectioning, box marks region shown in this figure. **B**: Ventral view of ganglia, digestive system, and retractor muscle. Nerves largely omitted. **C**: Dorsal right view of anterior cns and details of the cerebral innervation. Pedal nerves transparent. Scale bars: all 100 μm. Abbreviations: 1, double root of rhinophoral nerve; 2, presumed pleuro-pedal connective branching from ‘visceral loop’; 3, cerebropedal connective; 4, double connectives to optic ganglion; ag, accessory ganglia; apg, anterior pedal glands; bb, buccal bulb; bg, buccal ganglia; bn, buccal nerve; cpg, cerebropleural ganglion; dg, digestive gland; ey, eye; gc, bilateral ‘giant cell’ on headshield nerve; hn, headshield nerve; lag, accessory ganglia of headshield nerve; ln, labiotentacular nerve; lnag, accessory ganglia of labiotentacular nerve (more anterior); lpag, left parietal ganglion; med, medullary core of cerebropleural ganglion; mo, position of mouth opening; mu, ventral retractor muscle, note fused part between pedal and visceral ganglion; ogl, oral gland; opg, optic ganglion; opgn, nerves of optic ganglion; pg, pedal ganglion; pnd, dorsal pedal nerve; rhn, rhinophoral nerve; rnag, accessory ganglia of rhinophoral nerve (more posterior); rpag, right parietal ganglion; sc, statocyst; sgl, salivary gland; subg, subintestinal ganglion; supg, supraintestinal ganglion; vg, visceral ganglion; vn, visceral nerve(s); vl, ‘visceral loop’.

Examined individuals of *Helminthope psammobionta* were between 1 and 3.5 mm long and roughly circular in cross-section, with a diameter of 80 to 100 μm in extended specimens to nearly 200 μm in a contracted 1.5 mm specimen. The body is completely vermiform and lacks distinction of a head, foot, mantle cavity, or visceral sac (Figures 1 and 2). The head end is rounded and slightly wider than the rest of the body; it appears not to be fully retractable. The posterior end is ventrally flattened in crawling specimens. Specimens isolated in petri dishes crawl slowly (much slower than flatworms in the same sample but similar to certain nemertines) and move their body in a sinuous fashion, with the head moving from side to side. Disturbed specimens contract slightly, but curl up at the same time (Figures 1A and 3A’). Most major internal organs are visible in live specimens, especially ganglia, statocysts and spicules, given adequate illumination.

At least in histological sections, several body openings can be discerned. The mouth opens terminally on the snout but is hard to detect due to its small size. Two small ciliated pits (discernible only in histological sections) located at the sides of the tail indicate the caudal adhesive gland. The other body openings are strongly ciliated and located along the right body side: the genital opening at approximately one quarter, the nephropore at 2/5, and the anus at 4/5 of the total length (Figures 1A and 2).

**Figure 4:**
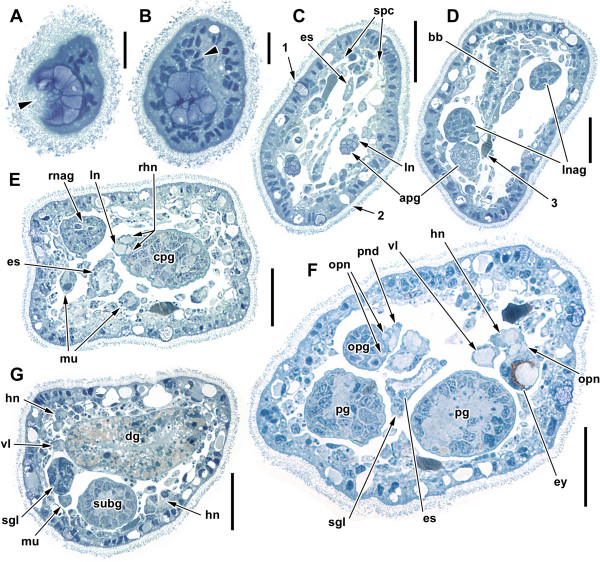
**Semithin cross-sections showing histological aspects of the head and nervous system of *H. psammobionta.*** Dorsal side to the upper right. **A**: Snout tip with opening of anterior pedal gland pad (arrowhead). **B**: Nuclei surrounding mouth opening (arrowhead) dorsal of mouth pad. **C**, **D**: Anterior head and various cell types (1-3). **E**: Front of cerebropleural ganglion (cpg). **F**: Posterior end of cpg and optic ganglion. **G**: Portion of visceral loop. Scale bars: **A**-**B**, 100 μm; **C**-**F**, 25 μm. Abbreviations: 1, pink-staining epidermal gland; 2, vacuolated epidermal gland; 3, amorphous cell; apg, anterior pedal glands; bb, buccal bulb; cpg, cerebropleural ganglion; dg, digestive gland; es, esophagus (thin portion); ey, eye; hn, headshield nerve; ln, labiotentacular nerve; lnag, accessory ganglion of labiotentacular nerve; mu, ventral muscle; opg, optic ganglion; opn, nerves to optic ganglion; pg, pedal ganglion; pnd, dorsal pedal nerve; rhn, rhinophoral nerve (double roots); rnag, accessory ganglion of rhinophoral nerve; sgl, salivary gland; spc, spicule cells; subg, subintestinal ganglion; vl, visceral loop.

The epidermis is strongly ciliated all around, with multiciliated cells, which are slender and contain a large and tall nucleus. Additionally, there are at least two distinct types of glandular cells: one type is barrel-shaped and filled with densely packed globules of pink-staining secretion, the apical opening wide and irregular (‘1’ in Figure 4C). The other type is very numerous and almost spherical (with a flattened, basal nucleus and large, clear or sometimes homogeneous grey vacuole opening through a terminal pore) (‘2’ in Figure 4C).

Below the epidermis, a variety of distinct cells surrounds the body cavity that contains the internal organs. One type of cell is largely oval and filled with numerous round blue droplets. Another type is large, amorphous and filled with a homogeneously stained, dark grey substance (‘3’ in Figure 4D).

In sections, spicule cells are discernible by the transparent spicule cavities enclosed by an irregular cell wall. They are located just beneath the epidermis (Figure 4C). The spicules are bent at an angle of approximately 160°; the cell’s nucleus is positioned inside this bend. Judging from live photographs, the well-visible spicules have a corrugated surface, especially towards their tips. Spicules are largely sorted at an angle of 45° to the longitudinal axis of the body.

The anterior digestive tract is flanked by paired anterior ‘pedal’ glands (pink-staining duct and lighter posterior part with widely-spaced nuclei) that open just ventrally to the mouth within a pad-like structure (see Figures 1A, 3B and 4A,B).

**Figure 5:**
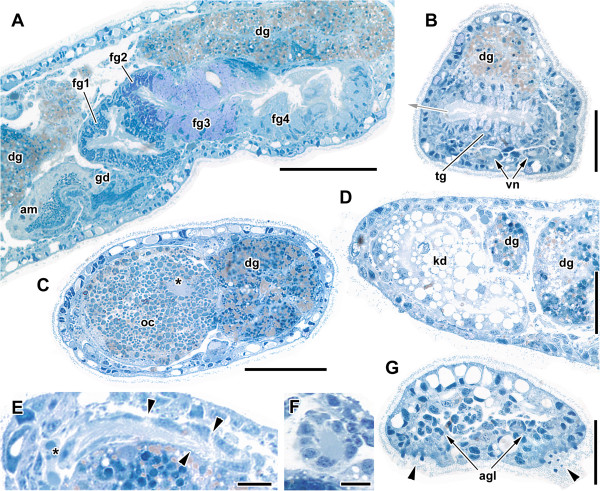
**Semithin sections showing histological aspects of the posterior half of the body of *H. psammobionta. *A**: Longitudinal section of reproductive system showing female glands. Anterior at right. **B**: Cross-section close to genital opening (grey arrow) and terminal gland. **C**: Yolky oocyte, nucleus indicated by asterisk. Dorsal at right. **D**: Kidney. Dorsal at left. **E**: Filter zone of kidney, sectioned longitudinally. Asterisk highlights nucleus of filter cell, arrowheads mark thin parts of wall. Dorsal at left. **F**: Cross-section through filter zone. **G**: Tail end showing ciliated openings of caudal adhesive gland (note emerging blue ‘pegs’, arrowheads). Scale bars: **A**-**D**, 50 μm; **E**-**F**, 10 μm; **G**, 25 μm. Abbreviations: agl, nuclei of adhesive gland cells; am, ampulla; dg, digestive gland; fg1-fg4, nidamental glands (proximal to distal); kd, kidney; oc, oocyte; tg, ‘terminal’ gland; vn, visceral nerve.

The caudal adhesive gland consists of a horseshoe-shaped cluster of cells in the posteroventral part of the tail. The gland opens through paired ciliated depressions on the lateroventral sides of the tail (Figures 1A and 2). While the gland’s cells themselves are difficult to detect, the ciliated pits are characterized by small strings of blue-staining secretion that project from pores through the epidermis (Figure 5G). In the reconstructed specimen (Figure 1A), the tail end is damaged so that parts of the gland are missing.

Muscle fibers are stained bright blue and are associated with the basement layers of all epithelial organs. A conspicuous pair of muscles runs along the ventral midline; both muscle bundles are fused between the pedal ganglia and the visceral ganglion (Figure 3B). The fibers attach to the anterior pedal glands anteriorly, posterior, they run along the visceral cords and the paired visceral nerve.

### Digestive system

The digestive system consists of a histologically uniform anterior part with enlarged midpiece (called buccal bulb herein) and associated glands, followed by the tubular digestive gland which ends blindly close to the tail, and the ciliated intestine near the end of the body (Figures 1A and 2).

The anterior digestive tract (a derived esophagus; see [32]) is formed by a strongly ciliated epithelium of slender columnar cells filled with numerous unstained apical vacuoles, giving the epithelium a ‘spongy’ appearance (Figure 4D). The portion following the mouth is very thin (diameter 12 μm) before widening into the buccal bulb (laterally flattened, height approx. 60 μm) located just anterior to the cerebral nerve ring; the part following the bulb is thin again but remains histologically identical. The single, tubular salivary gland (approx. 400 μm long, 20 μm thick) is visible externally, it runs parallel to the esophagus. The posterior part of the gland consists of columnar cells containing dark violet-staining vesicles that surround a central lumen (Figure 4G). The anterior duct is so thin that is becomes undetectable along the anterior esophagus, so the exact position of its opening into the digestive tract remains unclear (Figures 1A, 2 and 3B).

The undulating digestive gland is the most voluminous organ and extends all the way to the tail end. It consists of tall columnar cells, each filled with numerous blue and fewer unstained vesicles, surrounding the unbranched central lumen. In the posterior right portion of the digestive gland there is a short sickle-shaped region of epithelium which lacks vesicles (the ‘stomach’ in *Rhodope*; [35]). From there, the ciliated intestine emerges and leads to the anus on the right body side, at about 4/5 of the total body length.

### Kidney

The excretory system consists of a proximal duct lying freely in the hemocoel and of the bag-like kidney (90 μm) which connects directly to the nephropore. There is no associated heart or pericardium. The anteriorly located proximal duct (about 70 μm long, 8 μm wide) consists of flat, multiciliated cells that surround a central lumen (‘filter zone’ in Figures 1B, 2 and 5E,F). Parts of the wall are thin but nevertheless distinct (indicating a strong basal lamina); bundles of long cilia reach down the duct towards the kidney. The kidney itself is characterized by a thickened, irregular inner wall with typical unstained, round vacuoles (Figure 5D). The kidney connects directly to the ciliated nephropore located at about 2/5 of the body length.

### Reproductive system

The genital system of *Helminthope* is hermaphroditic and monaulic, i.e. a simple duct with one terminal opening. It consists of the tubular gonad followed by the ampulla, then a succession of 5 histologically separate (nidamental = eggmass-forming) glands plus a terminal (spermatophore-forming?) gland close to the ciliated genital opening (Figure 5A,B).

The gonad is an undulated tube that extends from the tail end to approximately half of the body length. It is located below the digestive gland. In the examined mature specimens it is densely filled with a variety of gamete precursors and ripe gametes, there is no remaining discernable lumen. Large oocytes can sometimes be identified by their larger nuclei and accumulation of blue-stained yolk droplets, some eventually filling most of the gonad’s diameter. The examined specimens never contained more than three of these fully formed eggs. Spermatozoa and their precursors (spermatids) are conspicuous in possessing an intensely dark-staining respectively screw-shaped or teardrop-shaped nucleus. Clusters of spermatids were found mainly in the posterior half of the gonad; ripe spermatozoa in bundles of up to 20 are found further anterior.

Following the anterior end of the gonad and a piece of undifferentiated gonoduct (ciliated, with outer muscular layer), the ampulla is a widened part that is filled densely with ripe spermatozoa (Figure 5A).

Distal to the ampulla – at approximately half of the body length – the gonoduct wall is strongly glandular, forming five consecutive nidamental glands (Figure 5A). The first gland is a short, bag-like expansion of one side of the gonoduct, its cells show grainy vesicles staining dark blue. This is followed by a small gland 2 which shows similar grains but that stain dark violet. Gland 3 is relatively large and bulbous compared to the other glands, it stains homogeneously light pink. Gland 4 is shorter again and stains homogeneously light blue. Gland 5 is the largest; it also stains light blue but contains large interspersed cells with an unstained vacuole. Following a short piece of unmodified gonoduct, there is a final (terminal) gland which is barrel-shaped and contains columnar glandular cells with pale pink-staining vacuoles (Figure 5B). The ciliated gonopore opens at approximately 1/4 of the body length.

### Central nervous system (CNS)

**Figure 6:**
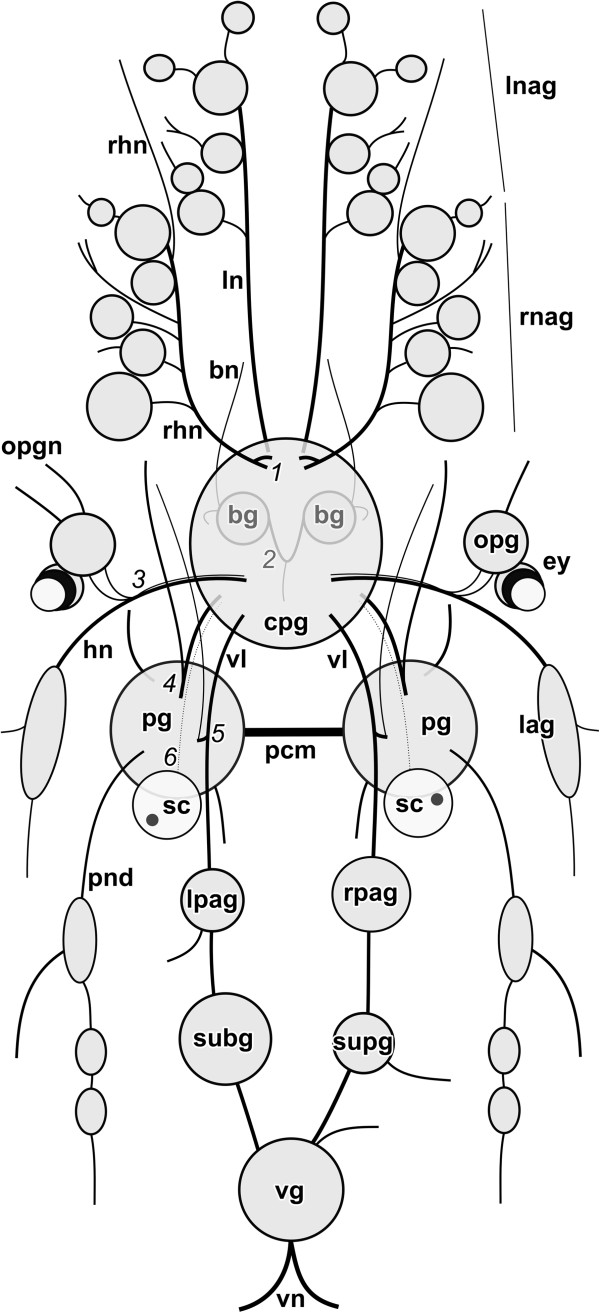
**Schematic dorsal view of the central nervous system of *H. psammobionta*.** Anterior side is up. Abbreviations: 1, double root of rhinophoral nerve; 2, buccal commissure with median nerve; 3, presumed headshield nerve with parallel nerve leading into double optic connectives; 4, cerebropedal connective with parallel static nerve and anterior pedal nerve at its base; 5, visceral loop with branch forming presumed pleuropedal connective; 6, static nerve running parallel to cerebropedal connective; bg, buccal ganglia; bn, buccal nerve; cpg, cerebropleural ganglion; ey, eye; hn, headshield nerve; lag, (lateral) accessory ganglia of headhield nerve; ln, labiotentacular nerve; lnag, accessory ganglia of labiotentacular nerve (more anterior); lpag, left parietal ganglion; opg, optic ganglion; opgn, nerves of optic ganglion; pcm, pedal commissure; pg, pedal ganglion; pnd, dorsal pedal nerve; rhn, rhinophoral nerve; rnag, accessory ganglia of rhinophoral nerve (more posterior); rpag, right parietal ganglion; sc, statocyst; subg, subintestinal ganglion; supg, supraintestinal ganglion; vg, visceral ganglion; vn, visceral nerve(s); vl, ‘visceral loop’.

The CNS of *Helminthope psammobionta* consists of the spherical cerebropleural ganglion (cpg), the paired pedal, buccal and optic ganglia ventral or lateral to the cpg and five ganglia on the very long visceral loop more posterior (Figures 2, 3 and 6). Numerous large accessory ganglia are associated with the nerves emerging from the cpg, smaller ones are found on a pedal and optic ganglion nerve. The eyes are located laterally and behind the optic ganglia; the large and conspicuous statocysts sit on the posterior sides of each pedal ganglion. All of these structures are visible in living specimens with transmitted light. Histological sections show that the cpg, pedal and buccal ganglia – and, to a lesser extent the visceral loop ganglia – contain a distinct central region formed by nerve fibers (medulla) and an outer cortex containing nuclei of neurons. In the other ganglia, neurons fill the entire ganglion evenly. All ganglia are enclosed in a homogeneous blue-staining cellular capsule that contains few flattened nuclei.

The most conspicuous and central element of the CNS is the almost spherical complex of fused left and right cerebral and pleural ganglia (the cerebropleural ganglion, cpg; diameter about 55 μm). Histologically, it is distinctly divided into cortex and fibrous medulla (see Figure 3C). The cerebral commissure remains detectable only from the wide median connection of the medullar mass. Remnants of the pleural ganglia are only detectable as an aggregation of neurons at the posterodorsal side of the cpg. Two pairs of thick nerves emerge from both the anterior and posterior faces of the cpg: from the anterior side the rhinophoral and labial nerves (Figure 4E), from the posterior side the headshield/optic nerves (Figure 4F, fibers almost fused, origin in the mediodorsal part of the medulla) and the combined visceral loop/pleuropedal connective. From the ventral side of the cpg emerge the thin cerebrobuccal connectives (more anterior) and the thick cerebropedal connectives (medioventral) besides the thin static nerve. Numbers used below follow the nomenclature by Staubach and Klussmann-Kolb [54] and Staubach [55].

The thick rhinophoral nerve (N3, diameter 5 μm) emerges from the anterior face of the cpg more dorsal than the labial nerve. The nerve shows two equally thick roots, one of which originates close to the root of the labial nerve (Figure 4E). The rhinophoral nerve runs anteriorly along the sides of the head and terminates near the mouth. Up to six pairs of accessory ganglia (diameter 10 to 20 μm; only 2 to 3 in small specimens) attach laterally to the proximal half of the nerve, either by direct contact or by a short branching anastomosis (Figure 3C). The accessory ganglia are spherical and full of neurons, the neuropil being limited to the fibers of the rhinophoral nerve (Figure 4E).

The equally thick labiotentacular nerve (N2) emerges from the cpg more ventrally and features, in its distal part, further (six) pairs of accessory ganglia. The more posterior accessory ganglia are thus innervated by the rhinophoral nerve, the anterior ones by the labial nerve (Figures 3C and 6). Thin nerves innervating the lateral epidermis of the snout are detectable in at least some of the accessory ganglia.

From the posterior face of the cerebropleural ganglion (anterior to the region with the presumed ‘pleural’ neurons) emerge the thick, paired headshield nerves (N4) (Figure 4F). The headshield nerves pass closely by the eyes on their way to the posterior flanks; each nerve features a single large, elongate ('lateral') accessory ganglion from which one nerve runs directly to the body wall, and another continues posteriorly (Figure 3A,C). This posterior branch is covered with neurons – some of them with a diameter of up to 10 μm – along much of its length and thus resembles a medullary cord.

The optic nerves run along the proximal part of each pleural nerve; the optic nerve then shows two connections to the optic ganglia (Figures 4F and 6). The spherical optic ganglia (Ø 15 μm) touch the anterior side of the eyes, but no direct nervous connection between the two could be detected. Each optic ganglion shows one, or possibly two additional medium-sized nerves that extend anteriorly.

The eyes face towards the sides (Figure 4F). Each eye consists of a spherical lens, followed by a cup-shaped pigment layer which is surrounded by a layer containing perhaps 5 or 6 nuclei (belonging to sensory cells?). The lens stains light grey/blue and is covered by a thin, apparently acellular but distinct blue membrane (a cornea?); the inner part of the lens shows a slight, irregular grey fringe (sensory microvilli?) (Figure 4F). The pigment layer consists of black or dark brown pigment granules. Some sections show a faint gap inside the pigment layer which might indicate that the pigment is contained within only two cells. The nuclei below the pigment cup presumably belong to the sensory cells of the eyes and the pigment cells; however, clear boundaries between the nuclei-bearing cells were not discernible in semi-thin sections.

Posterior to the headshield nerves emerge the paired visceral cords that connect to the ganglia on the visceral loop. The cords also appear to contain fibers of another origin, because after a short stretch a thick nerve branches off and connects to the posterodorsal side of the pedal ganglion (‘2’ in Figure 3C). Since there is no other connection between the cpg and the pedal ganglia except the more anterior cerebropedal connective, this connection should be the pleuropedal connective.

On the ventral side of the cpg, long and thin cerebrobuccal connectives emerge anteroventrally. The paired buccal ganglia usually lie more anterior and show two nerves: a paired one emerges from the base of each cerebrobuccal connective and runs along the buccal bulb (‘bn’ in Figure 3C); an unpaired nerve extends from the middle of the buccal commissure and extends posteriorly (‘2’ in Figure 6).

The paired pedal ganglia are the second largest ganglia (diameter 30 μm, 45 μm long). They are interconnected by the comparatively long pedal commissure, and together with the cpg form the cerebral nerve ring around the digestive tract. There are four connections: the paired cerebropedal connectives, and the presumed pleuropedal connectives that are present as short branches splitting off of the anterior portion of the visceral loop, approximately 50 μm behind the cerebropleural ganglion. From the bases of all connectives, thin (pedal?) nerves extend anteriorly. There are three further pairs of pedal nerves: one anterior, one posteriomedian, and one posterodorsal. The last pair extends along the flanks and features at least three small, ill-defined accessory ganglia similar to those found on the pleural nerves.

The statocysts are large, hollow spheres (∅ 15 μm) attached to the posterior face of each pedal ganglion (Figure 3B,C) and are enclosed in the same connective sheath. Each statocyst is formed by a wall of flat epithelial cells that the surround the fluid-filled lumen; there is a single spherical statolith. The presumed static nerve (a cerebral nerve) runs parallel to the cerebropedal connective, but is thin and could not be traced entirely.

The long visceral loop is untorted, i.e. euthyneurous. It features five widely-spaced ganglia – the most posterior one (the visceral ganglion) is located approximately 350 μm behind the cpg, or at one quarter of the body length. Both ganglia on the right visceral cord are located approximately 20 μm further anterior than their counterparts on the left cord (Figure 3A,B). The first pair of ganglia is separated from the back of the cpg and the front of the second pair by roughly 70 μm; the second pair is separated from the visceral ganglion by about 130 μm. The first two ganglia on the visceral loop are the left and right parietal ganglia; the right one is slightly larger (25 μm long vs. 20 μm), whereas the left one shows a thin posterior nerve (see Figures 6 and 3A). Both ganglia show at least two neurons that are larger than the others, and contain a large nucleus (but not ‘giant’ neurons). Second in order are the subintestinal (left) and supraintestinal ganglia (right); this time the left ganglion is larger (33 vs. 24 μm), but the right one shows a posterior nerve. The subintestinal ganglion contains two large neurons. The visceral ganglion is located medially, at the end of the visceral loop where the left and right visceral cords meet; the ganglion is about 45 μm long but elongate, it again contains two to three large neurons. A thin nerve emerges from the anterior right side, the thick visceral nerve emerges posteriorly. This conspicuous nerve splits into two equally thick branches just after leaving the ganglion (Figure 3A). Both branches run parallel along the ventral side of the animal (Figure 1A), and are accompanied by muscle fibers throughout their entire length. Judging from histological sections, the visceral nerves do not branch before terminating in the tail end, near (or in?) the adhesive gland.

## Discussion

*Helminthope psammobionta* is an extreme case among marine meiofaunal heterobranchs. It lacks almost all external characters that could identify it as a gastropod, and is one of the most aberrant free-living gastropods. Only the location of the genital, kidney and anal openings on the right body side are obvious remnants of the original gastropod body plan with torsion and resulting asymmetry. Without hard parts such as a radula and shell, only internal characters can help in evaluating the relationships of *Helminthope* to *Rhodope* (Table 2), and to other heterobranchs, from a morphological point of view. The original description [29] was based on characters that are visible in squeezed specimens observed under the light microscope (spicules, many ganglia, salivary gland – [29], BB, pers. obs.). Other parts of the animal (crucial connections between ganglia, nerves) needed higher resolution and superior scrutiny. Therefore, the original description of *H. psammobionta* could be corrected and supplemented considerably by combining histological investigation with 3D reconstruction of all major organ systems (see Table 1).

**Table 2:** **Comparison of divergent characters between *Rhodope *spp. and *Helminthope psammobionta***

	***Helminthope psammobionta***	***Rhodope *spp.**
Approx. length/width ratio (contracted - crawling)	8-25	3-9
Habitat	interstitial	littoral, interstitial (some both?)
‘Vesicle’ system	absent	present
Glands of the foot sole	lacking	generally present
Vestigial pharynx	not present	present
Anterior pedal = ‘oral’ glands	paired, tubular	paired, follicular (mixed with ‘true’ oral glands?)
Salivary glands	single, tubular	paired, follicular
Anterior lobe of digestive gland	lacking (or axial connection to esophagus)	extends beyond CNS
Position of intestine/anus	at 4/5 of body length, far from nephropore	at 1/3 of body length, close to nephropore
Form of kidney	sac-like, with proximal filtering duct	two thin branches with interspersed filtering knobs
Form of gonad	tubular, gametogenesis not spatially separated	2-3 posterior testicles, several anterior ovarial follicles
Number of ‘terminal’ glands in gonoduct	1	2
Eyes	with spherical lens, separate from cpg	with corpuscular lens, sitting
Rhinophoral nerve (double roots)	without basal ganglion, with large accessory ganglia	with basal ganglion, accessory ganglia small (or lacking?)
Labiotentacular nerve	undivided, with large accessory ganglia	bifurcated, accessory ganglia small or lacking
Postcerebral accessory ganglia	on ‘pleural’ nerves, also pedal nerves and possibly optic	none?
Separation of cerebral and pleural ganglia detectable	only internally	external fissures detectable in some species
Free visceral loop ganglia	5	1 (adult)
Scenario for ganglion nomenclature (parentheses indicate fusion)	(cg-plg)-1-2-3-4-5-(plg-cg)	(cg-plg-1)-(2–3)-(4-5-plg-cg) or (cg-plg-1-2)-3-(4-5-plg-cg)

Abbreviations as in Figures 2 and 6 except: cg, cerebral ganglion; plg, pleural ganglion.

### *Helminthope psammobionta* revisited - general histology

Haszprunar and Künz [41] compared ultrastructural characters of both described rhodopemorph genera, concluding that *Rhodope* showed similarities to doridoidean nudibranchs (epidermal cells with typical vacuoles, vesicle ‘network’ system, possession of verrucose spicules), while *H. psammobionta* lacked these characters, supporting the author’s notion that both genera were not closely related. Histology does not permit identification of the diagnostic epidermal vacuoles, but confirms that *Helminthope* lacks the enigmatic ‘vesicle system’. Another difference between both genera was the ‘parenchymatic’, compact body cavity detected in *Helminthope*[41]; this is not apparent from our histological examination – spacing of cells may be closer in *Helminthope* due to its smaller body diameter, but we conclude here that there is no fundamental difference in the body cavity of *Rhodope* species. We were not able to correlate the conspicuous amorphous ‘grey patch’ cells (Figure 4C,D) found in our material with Haszprunar and Künz’s results. Salvini-Plawen [29] mentioned subepidermal ‘platelet-like’ elements. No equivalent to these were evident in our sectioned material, although many epidermal glands show vacuoles that are visible as refracting bodies in live specimens.

### Anterior pedal and caudal adhesive glands

*Helminthope* possesses paired anterior glands (staining pink) that appear to be homologous to the equally pink-staining but diffuse and follicular glands mentioned for some *Rhodope* species (e.g., [56]). These were interpreted as ‘oral’ glands by previous authors [29,32]. None of the examined *Helminthope* specimens showed a connection of the glands into the digestive tract. Instead, one specimen showed a conspicuous patch (shown in Figure 4A,B) below the mouth opening through which the glands appear to open. Reinvestigation of an undescribed *Rhodope* species also shows that at least some of the diffuse pink-staining glands open at the sides of the head and not into the digestive tract (BB, pers. obs.). Therefore, we here regard these paired anterior glands not as oral glands, but as anterior pedal glands instead (see below). *Helminthope* lacks the single-celled glands that usually open through the foot sole of gastropods and can be detected as blue-staining bodies in histological examination (e.g. [57]). These glands are present along the ventral side of the body at least in *Rhodope rousei* Brenzinger, Wilson & Schrödl, 2011[32].

Salvini-Plawen [29] noted that *H*. *psammobionta* does not possess a caudal adhesive gland, separating it from *Rhodope* species. However, our results show that the gland is present. It is already externally visible in whole mounts stained with Safranin (BB, pers. obs.). Its cells are inconspicuous in histological sections, but the outline of the gland can still be reliably located by the presence of characteristic ‘pegs’ emerging from the cell’s apices, as is also the case in *Rhodope* (BB, pers. obs.). The cells histologically resemble the ‘normal’ unicellular pedal glands, but judging from their position may also be homologous to the posterior pedal glands of many basal heterobranchs [58].

Putative anterior and posterior pedal glands are present as distinct organ systems in many basal heterobranchs [51,58,59] but also more derived clades such as runcinaceans (*Ilbia* Burn, 1963 [60]), acochlidians or sacoglossans [22,57]. They generally open on top of the anterior pedal sole, and on the ventral side of the posterior foot sole, respectively. These glands are either paired or fused but open close together or via a common duct. The function of the posterior gland as an adhesive structure was observed in living *Helminthope* sp. from Belize: if disturbed, specimens attached themselves to the glass of a petri dish by the flattened tail end (KM Jörger, pers. comm.). Since the conspicuous paired visceral nerves terminate in/at the gland without anterior branching, the nerves may play a crucial role in controlling the adhesive mechanism but requires TEM study to investigate. Adhesive glands are convergently present in various meiofaunal organisms such as gastrotrichs, rhabdocoel flatworms and some annelids (e.g. [5,61,62]). Because these mechanisms commonly work with a double function (adhesive and detaching gland components), the double innervation of the tail end might indicate that this is the case also in rhodopemorphs.

### Digestive system

The digestive system of *Helminthope* is simplified compared to that of other gastropods, but is in principle identical to that of *Rhodope*. Histological characters are highly similar (BB, pers. obs.). Both genera lack an oral tube followed by the muscular pharynx with radula typical for gastropods. Instead, they possess a derived three-part esophagus that directly joins to the mouth opening and contains a novel ‘buccal’ bulb which functionally replaces a pharynx (see [32]). Both genera show a tubular digestive gland with a short intestine on the right body side. *Helminthope* differs from *Rhodope* in the marked elongation of the digestive tract (Table 2: buccal bulb is more elongate, there is no cephalic ‘caecum’ sensu [29], intestine and anus are shifted far posterior) and by having a single, non-follicular salivary gland. *Helminthope* lacks the small sac-like cavity into which the salivary glands open in *Rhodope* (argued to be a vestigial pharynx by [32]).

The peculiar single salivary gland of *Helminthope* is identifiable as such by histological characters (cells with dark blue-staining vesicles). The opening into the digestive tract could not be located in the examined material; it could never be traced further forward than the anterior part of the esophagus but should open far anterior, if interpretation of the anterior digestive tract as an esophagus is correct. The tubular form of the gland seems to be a result of less space in the body cavity due to body elongation. Judging from its slightly dextral position in histological sections, it might refer to the ancestrally right salivary gland. In *Rhodope*, the salivary glands are still paired, consist of numerous follicles, and (likely) open into the vestigial pharynx [32]. Salvini-Plawen [29] noted the gland’s visibility in live specimens but interpreted the gland to be a distal ‘genital tube’, thus locating the genital opening anteroventrally and misinterpreting other body openings (see below).

The three-part esophagus with vacuolate (and therefore elastic?) epithelium is a characteristic feature of rhodopemorphs. Its bulbous middle part was suggested to function as a sucking pump, aiding the ingestion of soft-bodied food [32]. Except for Riedl’s [34] successful table-top experiment in rearing littoral *Rhodope veranii* on a diet of the basal metazoan *Trichoplax* Schultze, 1883, there are still no direct observations of rhodopemorph feeding, as is often the case for micro- and meiofaunal gastropods. One specimen of *H. psammobionta* contained food remnants in the digestive gland but which resembled the general histology of the gland, indicating that food is soft to liquid. Candidates for food organisms found in the mesopsammon are large protists or metazoan eggs. Organisms feeding as ‘pump-suckers’ [6] are common among meiofaunal groups such as nematodes and gastrotrichs.

The digestive gland of *Helminthope* lacks a pronounced anterior-leading part (called ‘caecum’ by [29]) and is much more elongate but otherwise similar to that of *Rhodope* (Table 2). Riedl ([35]: Figure 23) observed the development of two digestive gland lobes from the stomach in young *Rhodope*, the anterior lobe extending beyond the opening of the esophagus. Salvini-Plawen [29] correctly noted that the connection of esophagus and digestive gland in *Helminthope* is axial (‘without anterior caecum’). The anterior lobe is either not developed in *Helminthope*, or the esophagus opening is simply shifted more anterior as a result of general body elongation.

In gastropods, the stomach is defined as the area into which the esophagus enters and from which the intestine exits; lobes of the digestive gland branch from in between [18]. Riedl [35] observed that in *R. veranii*, the ring-shaped larval stomach remains as a sickle-shaped zone surrounding the proximal intestine, close but not connected to the posterior end of the esophagus. This ‘stomach’ can be reliably distinguished from the surrounding digestive gland by the lack of blue- and yellow-staining vesicles, as in *Rhodope*[32]. In *Rhodope*, stomach, intestine and anus are located close to the nephropore early in ontogeny ([35]: figs. 13,15). In *Helminthope*, they are far from the nephropore and located back in the animal. We speculate that in the latter the anus is formed only after some body elongation takes place, thereby effectively relocating the stomach and anus (but not the otherwise associated nephropore) towards the tail.

Reductions of the digestive system make comparison to other basal heterobranchs difficult. Murchisonellidae are known to possess a unique ‘jaw apparatus’ and an apparently reduced pharynx [63]. *Henrya* Bartsch, 1947 also possesses a simple, long esophagus [64], *Koloonella* Laseron, 1959 species possess a peculiar glandularized esophagus (BB, pers. obs.). A three-part esophagus with ‘spongy’ epithelium at least in the midpart – possibly similar to that of rhodopemorphs – is mentioned e.g. for the valvatoid *Cornirostra* Ponder, 1990 [65,58]: p. 25]. The presence of a ‘derived’ esophagus is noted for different basal heterobranch lineages [13,14]. This may imply a more widespread phenomenon that is secondarily lost e.g. in limnic *Valvata* O.F. Müller, 1774 (according to [58]) and the architectonicoid *Omalogyra* Jeffreys, 1859 [59], genera that grouped as a monophylum in the study by Dinapoli and Klussmann-Kolb [13]. In the marine valvatoidean *Hyalogyrina* Marshall, 1988 [51: fig. 12], the esophagus shows a histology similar to rhodopemorphs but also possesses folds not present in the latter.

### Excretory system and lack of a heart

Salvini-Plawen originally described the kidney of *Helminthope* to be a ‘protonephridium’ positioned ‘about 100 μm behind the visceral ganglion’ [29: p. 307]. This fits with our results which indicate that the kidney contains two distinct parts: a proximal duct with multiciliated cells forming a ciliary flame and histologically distinct basal membrane, and a distal part with the diagnostic vacuolated epithelium. This implies that the proximal duct may function as a filter, with modification of the primary urine taking place in the vacuolated part. In *Rhodope*, the peculiar kidney has gained much attention due to its marked similarity to the branched protonephridium of flatworms (one of the factors thought to question its molluscan affinities; [31,36]). In contrast to *Helminthope*, this kidney consists of two ducts that extend along the right body side and converge at the nephropore; the ducts show the typical kidney epithelium and contain multiple interspersed filtering ‘knobs’ with a ciliary flame. According to Haszprunar’s [66] ultrastructural examination of *R. transtrosa* Salvini-Plawen 1991, these ‘pseudo-protonephridia’ lack the diagnostic basement membrane with ultrafiltration weir (only free hemocoelic rhogocytes possess this prerequisite for ultrafiltration). Given the data on other groups, the branched kidney of *Rhodope* looks more derived from a hypothetical ancestor than that of *Helminthope*. These differences could be attributable to the form of the body and body volume to surface ratios – the thicker body of *Rhodope* species may need a larger number of filters than the thin body of *Helminthope*.

The excretory organ of *Helminthope* resembles the paired larval/juvenile nephridia described recently in the chiton *Lepidochitona* Gray, 1821 [67,68]: these possess ‘larval’ protonephridia (with filter zone and vacuolated part) that become fully reduced, and ‘early adult’ protonephridia with an originally similar morphology that later becomes modified to form a metanephridial system after joining the pericardium. We assume that this mechanism is similar in heterobranch gastropods, including rhodopemorphs that possess only the (right) kidney as adults. Therefore the right-side asymmetry of the excretory system in *Helminthope* is consistent with a paedomorphic condition of an ‘early adult’, i.e. protonephridial-stage, nephridium.

Loss of the metanephridial system otherwise present in adult mollusks is related to the complete loss of the heart (and pericardium); for rhodopemorphs not any trace has been reported even for ontogenetic stages [31,32,35]. Lack of a heart was also described for some other small-bodied heterobranchs such as some acochlidians or the mesopsammic sacoglossan *Platyhedyle* Salvini-Plawen, 1973 [22,69], but a heart was later confirmed at least for the former [24]. Other presumably ‘heart-less’ gastropod taxa are the ‘allogastropod’ *Cima* Chaster, 1896 (according to [70]) and the sacoglossan *Alderia modesta* (Lovén, 1844) [71]. These species, however, possess a ‘normal’, i.e. sac-like kidney. Therefore, rhodopemorphs are unique even among other heart-less gastropods in possessing a special protonephridial-like excretory system which resembles a protonephridial-stage adult kidney.

### Reproductive system

Characters of the reproductive systems are considered to be of major systematic value in heterobranchs [72-74], and many anatomical descriptions include detailed accounts of these organs. *Helminthope psammobionta* is a simultaneous hermaphrodite with an unbranched (= monaulic) genital system. Unusual for a hermaphrodite, there are no obvious structures for the storage of received sperm (‘allosperm receptacles’).

Our examination shows some differences in organization compared to the original description by Salvini-Plawen ([29]; see Table 1). In consequence, the reproductive system is not fundamentally different from that of *Rhodope* (see [32]). Differences include the organization of the gonad: in *Rhodope* it is ramified with posterior testicles and more anterior ovarial follicles [31,32,56]. There appear to be no separate regions of gametogenesis in *Helminthope*, oocytes equipped with yolk are located along much of the gonad, but appear to be relatively smaller than those of *Rhodope*. Spermatozoa show the corkscrew-shaped head typical for heterobranchs [75-77], but without TEM data comparison to heterobranch subgroups is not possible.

The nidamental gland mass consists of five separable glands in *H. psammobionta* and also *R. rousei*[32]. Other *Rhodope* species examined here show at least four nidamental glands (BB, pers. obs.). This is a higher number than in most other heterobranchs which are in most cases described with only three types of gland (see [78,79]). Therefore it is difficult to homologize the glands in rhodopemorphs.

Contrary to *Rhodope* species, *Helminthope* possesses only a single ‘terminal’ gland (Table 2). According to histological characters, the gland in *Helminthope* is homologous to the proximal of two terminal glands in *R. rousei* ([32]: ‘barrel-shaped’ gland) and other *Rhodope* species ([31], BB pers. obs.). In *Helminthope*, the gland is more elongate and less regular on a cellular level; also, it is separated from the last nidamental gland by a comparably longer piece of undifferentiated gonoduct. Some other basal heterobranchs (e.g. the orbitestellid *Microdiscula* Thiele, 1912, see [80]), possess prostate tissue distally to the nidamental glands, i.e. in the same position as the terminal gland(s). Because a copulatory organ located more anterior is lacking in rhodopemorphs, these glands were hypothesized to form spermatophores (see [32]). In contrast to *Rhodope* specimens that were repeatedly observed to contain free spermatozoa within the body cavity [31,32,66], our results and previous TEM studies [9,41] did not confirm this phenomenon, which is associated with hypodermal insemination, in *Helminthope*.

The reproductive system of the murchisonellid *Henrya* is depicted as monaulic and includes two seminal receptacles and a cephalic copulatory organ close to the head [64]. Nothing is known about the other supposed murchisonellids.

### Central nervous system

The nervous system of *Helminthope psammobionta* is unique among gastropods in its scattered arrangement of ganglia (involving five distinct ganglia on the visceral loop and numerous ‘accessory’ ganglia). This is contrasted by the fusion of cerebral and pleural ganglia to an almost spherical structure. All these ganglia can be externally localized in living specimens via a light microscope ([29], KM Jörger, pers. comm.). Contrary to the original description, we were able to identify additional accessory ganglia posterior to the cerebropleural ganglion, and an extended set of nerves next to minor differences such as the anterior, not posterior position of the buccal ganglia (see Table 1).

Nervous system characters have traditionally and frequently been employed to define heterobranch relationships (e.g. [30,81], but see [82]). Especially higher taxa such as the Euthyneura = Pentaganglionata are by their name defined by nervous system characters, i.e. the untorted state of the visceral loop or the presence of five distinct ganglia on it during ontogeny. The recently recovered more basal position of rhodopemorphs, outside Euthyneura, leads to the question how and when ‘typical’ heterobranch nervous system features evolved, i.e. the aforementioned characters, the considered diagnostic set of cerebral nerves with double cerebro-rhinophoral root, or the sensory Hancock’s organs.

### Cerebral nerves

The cerebral nerves have gained considerable attention in defining major taxa among Heterobranchia (e.g. [81,83,84]). Their correct identification is regarded as relevant for understanding questions about evolutionary patterns within Heterobranchia and their currently assumed sistergroup, the Caenogastropoda: which nerves are homologous between larger groups, how complex was the ‘ancestral’ pattern, and how did the nerves evolve? According to Huber [81], cerebral nerve complexity increases from caenogastropods to opisthobranchs, although ‘derived’ pulmonates have rather simple, i.e. plesiomorphic nervous systems. After Nordsieck [83], however, the ancestral euthyneuran already possessed a full set of nerves. Recent topologies with para- or polyphyletic opisthobranchs [13,14] imply evolution of a secondarily simple set of cerebral nerves in pulmonates, with still unclear homologies of the remaining nerves.

Heterobranchs possess several pairs of sensory cerebral nerves [81, terminology after 54–55]. The ‘typical’ set involves paired static, optic, oral (N1), labiotentacular (N2), rhinophoral (N3) and ‘headshield’ nerves (N4). Except for the first two pairs all nerves innervate larger areas of the epidermis, especially head appendages when present. It should be noted that in many taxa there is a lower number of nerves, which implies fusion or loss. Therefore, assumptions of homologies are not easy to evaluate, and nerves may have been confused frequently.

Our material of *Helminthope psammobionta* shows candidates for at least five of the six aforementioned cerebral nerves emerging from the cerebropleural ganglion (cpg). Static and optic nerves are present, as would be expected from a species that possesses statocysts and eyes. The oral nerve (N1) is either missing (due to reorganization of the anterior digestive tract?), or alternatively incorporated either into the thick labiotentacular or rhinophoral nerves (N2 and N3). The N2 is characterized by its anteroventral position in the cpg, and because it innervates the anterior sides of the snout. This area is, in rhodopemorphs, considered equivalent to the ‘anterior portion of the Hancock’s organs’ [29,40], distinct epidermal sensory areas found at the sides of the head of many heterobranchs (e.g. [55]). In contrast, the rhinophoral nerve (N3) is more dorsal, possesses widely separated double roots (one emerging next to the labiotentacular nerve, but see below), and mainly innervates the posterior sides of the snout. The thick nerve based in the ‘pleural’ portion of the cpg and running parallel to the optic nerve might either be the headshield nerve (N4, nervus clypei-capitis) or a ‘pleural’ nerve, i.e. emerging from the pleural portion of the cpg. We prefer the first interpretation, since pleural nerves are generally lacking in normal-sized, i.e. small heterobranchs [85], but a N4 is found in some [81].

This set of cerebral nerves conforms well to that of *Rhodope* but shows distinct differences. The optic nerve of *R. veranii* was described to split off ventrally of the pleuropedal connective [40], and Salvini-Plawen [29] noted it to emerge from the ‘terminal cerebropedal’ = pleuropedal connective also in *H. psammobionta*. Neither is the case in our material of *Helminthope*, where the nerve emerges dorsolaterally, close to but distinct from the putative N4.

There are some differences to the nerves found in *Rhodope*. The N2 = labiotentacular nerve of *Rhodope* is basally forked, in contrast to *Helminthope*, but resembling the condition found in caenogastropods, some ‘allogastropods’, i.e. architectonicoids or valvatoideans (e.g. [17,51,58,86]) but also many euthyneurans, i.e. the cephalaspid *Haminoea* Turton & Kingston, 1830 (see [55]). The N3 = rhinophoral nerve is also double-rooted in *Rhodope*, but possesses a slender ganglion at its base [32,40]. In *Rhodope*, a possible equivalent to the N4 = headshield nerve is the strong ‘lateral’ nerve, although this nerve was described with double roots in the pleural and pedal ganglia [32,39,40]. In the same position, the nervous system of larval *R*. *veranii* shows distinct ‘cerebropleural’ nerves (the right one bifurcated) according to Riedl [35]. This nerve is possibly homologous to the ‘lateral’ nerve of adult *Rhodope* ([35]: fig. 15a) and innervates approximately the same area as the N4 in *Helminthope*.

### Double cerebral connectives

**Figure 7:**
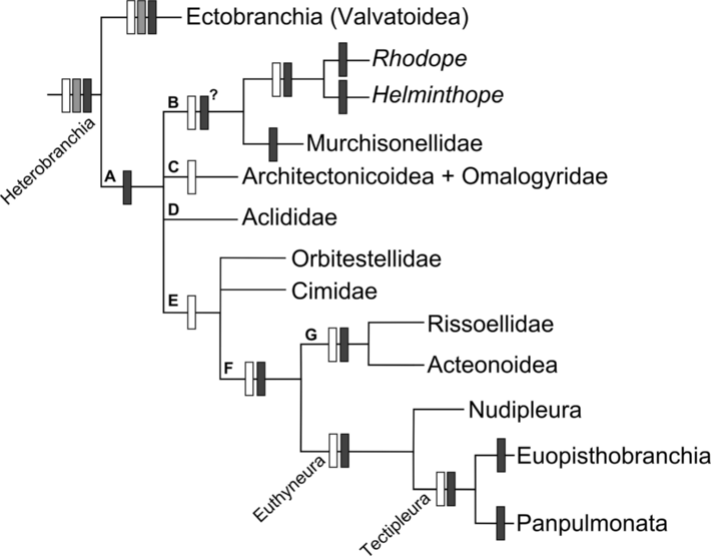
**Simplified consensus cladogram of Heterobranchia according to [**[13-15,116]**].** White boxes: clades with strong molecular support according to the aforementioned studies. Grey boxes: possible synapomorphies regarding sperm ultrastructure [75-77,110]. Black boxes: possible morphological synapomorphies (see text for further details). Heterobranch taxon sampling and apomorphies listed here are not exhaustive, and focused on taxa and characters relevant for discussing relationships with rhodopemorphs; reversals in subgroups are not indicated. Heterobranchia: spiral sperm, hyperstrophic larval shell, original gastropod ctenidium lost, pallial kidney, simultaneous hermaphroditism with ovotestis, loss of paraspermatozoa, among others [17]. Digestive system simplified: radular cartilages and esophageal pouches lost, paired buccal retractors [51]. Special arrangement of mitochondrial genes [117]. Ectobranchia: specialized ectobranch gill, paired pallial tentacles, sperm characters [51]. Node A: ciliary tracts present in mantle cavity; gill, jaws lost (?) Early development of 4d-mesentoblast (?). Node B: pharynx reduced; esophagus vacuolated (?). Rhodopemorpha: body wormshaped, meiofaunal syndrome characters (e.g., loss of body appendages and mantle cavity; presence of caudal adhesive gland, accessory ganglia, spicules); euthyneurous, pentaganglionate nervous system with double-rooted rhinophoral nerve; esophageal pump present/pharynx vestigial or lost; protonephridial-stage kidney retained in adults, among others ([32], this study). Nodes C,D,E: unknown. Node F: Giant neurons (in macroscopic members), possibly pentaganglionate condition (at least in early ontogeny). Euthyneura: Euthyneury (several reversals in subgroups), pentaganglionate CNS at least during ontogeny (?), rhinophores (?). Euopisthobranchia: esophageal gizzard with cuticle [14]. Panpulmonata: double-rooted rhinophoral nerve (?).

Double connectives between the cerebral ganglion and one of the thick cerebral nerves (called rhinophoral nerve, N3 herein) were considered to be a feature diagnostic of some higher heterobranchs [40], namely opisthobranchs and Pyramidelloidea. A double connective in this nerve is also found in rhodopemorphs ([32,40], this study), which would thus support placement with traditional opisthobranchs and/or Pyramidellidae Gray, 1840. In pulmonates, the so-called procerebrum (a neurosecretory structure characterized by ‘globineurons’) also possesses double roots [87,88]. Jörger *et al.*[14] recovered a mix of the aforementioned clades among Euthyneura and therefore indicated both double rooted structures – rhinophoral ganglion and procerebrum – to be potentially homologous, although this possibility was earlier disregarded due to histological and ontogenetic differences (e.g. [40]). These differences may, however, not affect the presence of a double root. In more recent studies, double rooted ‘rhinophoral’ ganglia were found in rhodopemorphs (not Euthyneura according to preliminary molecular data, [44]) and, inside Euthyneura, so far only among panpulmonate pyramidelloids, ‘opisthobranch’ sacoglossans and acochlidians [47,53,81]. Several other panpulmonates possess the neurosecretory procerebrum with double roots (see [40,86]). We are not aware of further records of double connectives among the remaining Euthyneura or acteonids, and only few euopisthobranchs have been indicated to possess the double connective, i.e. *Runcina*[81] and possibly *Pluscula*[26]. It remains unclear whether these double roots *per se* are homologous, since it is so far not clear which nerve tracts originally fused (or divided) to form the double roots; ontogenetic data on this particular phenomenon are entirely lacking. However, different nerves of the aforementioned ‘basic’ set were suggested to play part in the double root: some examples are the putative inclusion of nerves N3+4 in the sacoglossan *Elysia* Risso, 1818, *Gascoignella* Jensen, 1985 or *Platyhedyle* ([81]: p. 400], [22,53]) or the N3 + optic nerve in some acochlidians [14,25,47,52]. In *Helminthope*, one root of the N3 emerges close to the N2, therefore the double-rooted N3 may be product of partial fusion of fibers of N2+3, or one root may have originated from the otherwise missing N1. If rhodopemorphs are basal heterobranchs, as indicated by molecular data, then the double roots evolved convergently to those of panpulmonates (see Figure 7). Counter to our *a priori* homology assumption, which was based on criteria of structure and relative positions, an origin of rhodopemorphs among lower heterobranchs may also support an alternative scenario. The innermost cerebral nerve could refer to the N1, and the thicker, double-rooted cerebral nerve of *Helminthope* could be a fused N2 and N3. This possibility needs to be evaluated in the light of clarifying the identity and homology of bifid “tentacular” nerves of caenogastropods and “lower” heterobranchs versus “higher” heterobranchs often having separate cerebral N1-4.

### Accessory ganglia

Salvini-Plawen [29] described *Helminthope psammobionta* to possess ‘two complexes of accessory ganglia’ anterior to the cerebropleural ganglion (his Figure 4 shows approximately 5 pairs of ganglia), and assumed them to be associated with the cerebral nerves. We can show that these anterior ganglia are innervated by the putative labiotentacular nerve (N2) and the more posterior ones by the rhinophoral nerve (N3). The number of accessory ganglia appears to vary between individuals; some possess less than the 12 pairs shown in Figure 6.

Accessory ganglia on the same nerves are known for at least some *Rhodope* species ([40], BB, pers. obs.) but are always rather inconspicuous in histological sections. Accessory ganglia on the N2 and N3 are known for the majority of meiofaunal slugs (e.g. [7,22,25,81,89]), and, in combination with otherwise regressive features, also are typical of the ‘meiofaunal syndrome’. In short-headed taxa such as acochlidians the ganglia form a large, compact mass. More similar to the condition found in *Helminthope*, the nudibranch *Pseudovermis* Périaslavzeff, 1891 appears to possess numerous smaller ganglia along the sides of its ‘acorn-shaped’ snout [81,89]. Since the ganglia are supplied by sensory nerves, they were argued to be part of an enhanced sensory apparatus, facilitating food detection or path finding among three-dimensional interstitial pore spaces [26].

*Helminthope* is so far the only known microslug that possesses accessory ganglia also behind the cerebral nerve ring. These postcerebral accessory ganglia are innervated by at least one of the pedal nerves, possibly the additional nerve of the optic ganglia, and most prominently the headshield nerve. All these ganglia appear to innervate the flanks of the anterior body half and are elongate instead of spherical.

The formation of accessory ganglia in rhodopemorphs is correlated to the fact that many larger nerves contain nuclei/neurons along their length, giving the impression of medullary cords [29,40]. Due to the elongation of the body and nerves in *Helminthope*, the formation of additional ganglia may be necessary for fast processing of signals.

### Sensory organs

The eyes of *Helminthope psammobionta* show a spherical, solid lens, as usual in gastropods [18]. *Rhodope* species characteristically possess a lens made up of discrete bodies and seem to lack a cornea [31,32]. Therefore, *Helminthope* presumably shows the ancestral eye type, whereas the corpuscular lens of *Rhodope* appears to be an autapomorphy of the genus. At least one *Helminthope*-like rhodopemorph lacks eyes (MS, pers. obs.), which is not unusual for meiofaunal taxa [7].

The optic ganglia of *Helminthope* are large (compared to the eyes) and possess an additional nerve that runs along the flanks. This nerve is presumably the reason for the presence of double connectives of the optic ganglion, indicating that the ganglion is a product of fusion. Double cerebro-optic connectives are otherwise known for the acochlidian *Strubellia* Odhner, 1937 [52]; there, an additional nerve of unknown function connects to a branch of the rhinophoral nerve. The optic ganglia of *Rhodope* were described to be cup-like structures embedding the eyes [32,40]. Given the present results, the cells in *Rhodope* may alternatively be the sensory cells of the eyes as in *Helminthope*, and not a ganglion per se.

Statocysts are conspicuous elements in the CNS of *Helminthope* and *Rhodope*. They are large (compared to the body diameter) in *Helminthope*, but middle-sized to small in *Rhodope* species [32,40]. The presumed static nerve could not be followed along all of its length in our material and was not mentioned for other rhodopemorphs.

Epidermal sense organs such as Hancock’s organs on the sides of the head or an osphradium on the right side are not detectable in *Helminthope*. However, the presence of accessory ganglia on sensory nerves in the sides of the snout indicates that equivalents of the former might be present. A chemosensory osphradium, innervated by a nerve of the supraintestinal ganglion, was indicated for larvae (but not adults) of *R. veranii*[35]. *Helminthope* possesses a ‘supraintestinal’ nerve, but no apparent associated organ.

### Visceral loop ganglia and nerves

Salvini-Plawen [29] described the expanded pentaganglionate and euthyneurous visceral loop of *Helminthope psammobionta* and named the five free ganglia (from front to back) as the left and right parietal ganglia, the sub- and supraintestinal ganglia, and the visceral (=abdominal) ganglion. We follow the same interpretation here.

*Helminthope* varies greatly from *Rhodope* which possesses only a single free ganglion on the comparatively short visceral loop. This ganglion was considered to be a fused subesophageal and visceral ganglion [32,35] or simply the visceral ganglion [40], the remaining ganglia of the loop being joined anteriorly to the cerebropleural ganglia (see Table 2). The visceral loop of *Helminthope* resembles that of larval *Rhodope*[35] in possessing a true pentaganglionate condition with five unfused ganglia. *Helminthope* is therefore one of the few known heterobranchs to possess five free ganglia as an adult (see below), but is not part of the current Euthyneura = Pentaganglionata according to preliminary molecular results. Salvini-Plawen [29] gave phylogenetic emphasis to the left position of the visceral ganglion in rhodopemorphs, however, lies in a median position.

Our material of *Helminthope* shows nerves only on the left parietal, supraintestinal, and visceral ganglia. Riedl [35] identified nerves in the sub- and supraintestinal ganglia plus two strong nerves emerging from the visceral ganglion. Salvini-Plawen [29] did not show nerves of the visceral loop ganglia except for the paired visceral nerves. He found traces of streptoneury in the nerve fibers of the visceral ganglion that lead into the visceral nerves; we were not able to confirm this.

The visceral nerve of heterobranchs usually is a single strong nerve innervating the inner organs of the visceral sac. In rhodopemorphs there are two equally thick branches that run along the ventral side of the body and terminate near the caudal adhesive gland (this study, [32]). This unusual presence of two nerves instead of one might indicate that the nerves and the ganglion are a product of fusion, which is reflected in the confused nomenclature found in previous studies. In *Helminthope* the nerves split just behind the ganglion ([29], this study); originally, the right nerve was called the visceral nerve and the left one a ‘genital nerve’. In *Rhodope veranii* and *R. transtrosa*, the nerves even appear to originate partly in both the more anterior ganglia and the sides of the visceral ganglion, indicating fusion of ontogenetically separate nerves. Accordingly, Haszprunar and Huber [40] identified the left branch as a ‘genitovisceral’ nerve, and the right one (with more obvious partial root in the supraesophageal ganglion) as a ‘pallial’ nerve. In *R. rousei*, both nerves show at least some fibers that originate outside of the visceral ganglion [32]. On the other hand, the paired visceral nerves originate directly in the visceral ganglion in larval *Rhodope*[35], as they do in *Helminthope*.

The presence of five visceral loop ganglia in rhodopemorphs is of considerable phylogenetic interest. As stated by Schrödl et al. [15], rhodopemorphs are Heterobranchia that are pentaganglionate and euthyneurous but fall outside the current concept of the taxon Pentaganglionata = Euthyneura (*sensu lato*, including Acteonoidea). This leads to three possible scenarios: 1), the pentaganglionate condition evolved earlier than thought, i.e. at least in the last common ancestor of rhodopemorphs and euthyneurans, but was lost independently or not yet detected in intermediate (paraphyletic) ‘basal’ heterobranch taxa, 2), the pentaganglionate condition evolved convergently among rhodopemorphs and euthyneurans, or 3), the phylogenetic position of rhodopemorphs (outside of Euthyneura) recovered in molecular studies is wrong.

The taxonomic importance of the visceral loop configuration lies in the considerable attention it gained as a means to delineate major taxa. Inspired by Schmekel [90], Haszprunar [17] created the taxon Pentaganglionata to include all heterobranchs with five ganglia on the visceral loop at least during some point in ontogeny, as opposed to triganglionate heterobranch ‘allogastropods’ and all other gastropods. The additional (= left and right parietal) ganglia were presumed to be ‘derived from the pleural ganglia through elongation of the cephalopedal mass’ at an early point of ontogeny [17]. One can easily imagine this scenario of elongation to be the case in *Helminthope*.

However, only few Pentaganglionata have been observed to possess the namesake five ganglia at some point of their ontogeny (most possess fewer, but some even more than five, e.g. the ‘hexaganglionate’ *Chilina* Gray, 1828; see [82]), and is not clear if these ganglia represent homologous structures: a pentaganglionate visceral loop was reported for few members of all four major euthyneuran s.l. clades: in some *Acteon* species, ontogenetic stages of the nudipleuran *Aeolidiella* Bergh, 1867, in the euopisthobranch *Akera* O.F. Müller, 1776, and in the panpulmonates *Lymnaea* Lamarck, 1799 ([91-93], see [18,82]). Other taxa have been reported to lack five separate ganglia during their ontogeny (e.g. the panpulmonate *Ovatella* Bivona-Bernardi, 1832, [94]). In general, few species have been studied in sufficient histological detail and in sufficiently early larval stages to exclude the existence of a pentaganglionate stage. The presence and identity of potentially fused visceral loop ganglia in triganglionate systems remains to be tested by more sensible, e.g. immunocytochemical, techniques. It therefore remains unclear whether the pentaganglionate condition is homologous or even shared among Euthyneura (s.l.) and if yes, at which phylogenetic level (Ur-Euthyneura or elsewhere) it occurred for the first time. While the Pentaganglionata sensu Euthyneura hypothesis is rejected, we would not dismiss the possibility that the two additional, parietal ganglia on the visceral loop are an innovation of the last common ancestor of Rhodopemorpha and euthyneurans.

### Meiofaunal syndrome at an extreme

Meiofaunal slugs resemble small, unpigmented ‘worms’ that can be extracted from subtidal, well oxygenated sands (see [95]). Many species possess a set of typical characters (herein summarized as ‘meiofaunal syndrome’, [5-8]), aspects that are in this combination not found in small slugs that are not mesopsammic, e.g. the littoral runcinids or some progenetic nudibranchs (*Vayssierea* Risbec, 1928) and sacoglossans (*Limapontia* Johnston, 1836) [81,96,97].

*Helminthope psammobionta* is an exemplary meiofaunal slug that takes adaptations to the extreme: it shares with *Rhodope* the wormlike habit without body appendages, the strong ciliation, curved spicules, caudal adhesive gland, and accessory ganglia (see [32]). *Helminthope*, however, differs in its extreme elongation of the body (with parallel elongation, narrowing and simplification of internal organs) and complete loss of pigmentation (described *Rhodope* species are opaque white and may possess one or more colored bands; [42]). A still unexamined group of apparently mesopsammic rhodopemorphs with peculiar cross-shaped spicules (see [9,29]) is externally similar to *Helminthope* in habit (thread-like, unpigmented, with spheroid cerebropleural ganglia; BB, pers. obs.) and was indicated to represent a separate lineage [42]. Not all rhodopemorph species are meiofaunal, but they all show the morphological adaptations typical for interstitial sand-dwellers and appear well-adapted to interstitial life. Some coloured members of *Rhodope* may have recolonized (epi)benthic habitats, or may alternatively represent phylogenetically basal forms retaining plesiomorphic features.

Compared to other meiofaunal slugs, *Helminthope* externally resembles most closely the aeolid nudibranch *Pseudovermis*: both share the very elongate body and the slightly widened (‘acorn-shaped’) head presumably used as a wedge for digging [6]. *Pseudovermis* species, however, differ in the possession of more or less rudimentary dorsal body appendages (cerata, typical for aeolids), and internal organ systems of the genus are not as simple and paedomorphic/aberrant as in *Helminthope* but otherwise resemble other aeolids (e.g. [89,98]). No other free-living gastropods are similarly wormlike (judging from length/width ratios); only some endoparasitic eulimoid caenogastropods have similarly elongate, externally featureless bodies [99,100]. Among other meiofaunal metazoans, the almost threadlike habit is convergently found in particular ‘subsurface intertidal’ turbellarians [101], several nemertines, and lobatocerebrid worms that share their habitat with *Helminthope* [43, GH, pers. obs.].

### The role of paedomorphosis

Both the morphology of meiofaunal organisms and that of early Heterobranchia has frequently been associated with paedomorphosis, i.e. the retainment of larval or juvenile characters in the adult (see [102] for terminology). Alternatively, selection for small body sizes may simply lead to miniaturization [103], but not modification of adult morphologies. The idea that meiofaunal metazoans have largely evolved through such progenetic processes has been examined in particular for annelids (e.g. [103-105]). For Heterobranchia it has been assumed that the smallness and reduction of anatomical features found in many basal taxa were partly due to progenesis in the common ancestor [18,58]. Rhodopemorphs lack many typical heterobranch and general gastropod characters (e.g. those associated with the shell, mantle cavity, and pharynx). We hypothesize these reductions and the ‘larval’ organization of e.g. the visceral loop and the kidney to be indicators of progenesis.

Riedl’s [35] investigation of the ontogeny of *Rhodope veranii* is of particular importance for this: he showed that development (at least in the examined species) is unique but lacks a long-lived planktonic larval stage, which is quite typical for many microgastropods [58]. The hatching stage is a derived crawl-away larva of elongate drop-shaped appearance (called ‘Reisinger’ larva by Riedl [35]); it does not develop a shell (although a putative shell gland is present for a short time), operculum, or the cephalic velum otherwise typical for larval gastropods. Rhodopemorphs largely retain this ‘drop-shaped’ outer appearance after metamorphosis. Adult organ systems do not increase much in complexity during ontogeny and therefore appear paedomorphic. For example, the simple digestive system without a muscular pharynx and radula (which are usually developed late in ontogeny; [106]) and with only a short intestine (considered paedomorphic at least for patellogastropods; [107]) is similar to early ontogenetic stages. The tubular gonad and the unbranched gonoduct appear similar to the anlagen of these organs, i.e. paedomorphic: the former originates from a simple band of mesoderm (e.g. [48]), the latter is formed from a tubular invagination of ectoderm [106]. As discussed above, the configuration of ganglia in *Helminthope* (except for the accessory ganglia) is highly similar to what Riedl [35] observed in 13 days old *Rhodope*, with still unfused visceral loop ganglia spread along the longitudinal body axis. Also, the lack of a heart (in mollusks developed shortly before metamorphosis, [108]) and therefore presence of only a protonephridial-type kidney (present before the heart; [68]) are early ontogenetic characters persisting in the adult. While heterobranchs are hypothesized to have evolved from an apogastropod ancestor in the centimeter size range by progenetic miniaturization and simplification especially of digestive and mantle cavity organs [18], rhodopemorphs have reduced body complexity even further parallel to their invasion of meiofaunal habitats accompanied by progenesis. *Helminthope* is at the current meiofaunal syndrome and progenetic extreme.

What mechanisms cause *Helminthope* to be so extraordinarily elongate? There are currently no developmental data on early ontogeny of *Helminthope*, but comparison to developmental stages of *Rhodope veranii* described by Riedl [35] suggests that a large part of longitudinal extension in *Helminthope* takes place in an early stage of development, i.e. before the equivalent of larval stages found at day 10 to 12: at this point, larval *Rhodope* possess still unfused visceral ganglia on a long visceral loop, and the anus is not yet formed [[35]: figs. 13–16]. In *Helminthope*, a scenario with an early elongation (i.e. accelerated somatic growth or peramorphosis, [102]) would explain why ganglia on the visceral loop remain unfused and paedomorphic (the loop becomes stretched) and why the position of the anus is far posterior, separate from the nephropore (because it is only formed after considerable elongation of the body). We thus hypothesize that *Helminthope* originated from a stouter-bodied, more *Rhodope*-like ancestor by progressive progenesis coupled with peramorphosis (body hypertrophy) at an early ontogenetic stage, thus resulting in a habit partially resembling an over-elongate larva of already paedomorphic *Rhodope*. To test this hypothesis, ontogenetic data on *Helminthope* are required.

### Origin of Rhodopemorpha

The historical confusion surrounding the phylogenetic position of *Rhodope* – gastropod or not? Opisthobranch, or pulmonate, euthyneuran? – was most recently summarized by [42] and [32]. Rhodopemorphs are fascinating and highly unusual – they look like worms but are gastropods since they retain some aspects of the original gastropod torsion, i.e. the position of some body openings asymmetrically on the right. They are specifically heterobranch gastropods due to the spiral sperm heads, the epiathroid, euthyneurous and pentaganglionate nervous system [17], and other characters such as the ‘typical heterobranch’ mode of copulation and the form of the spawn [34].

*Helminthope* was originally described as part of Rhodopemorpha by Salvini-Plawen [29]. Later, its affiliation to *Rhodope* and rhodopemorph affinities to some spicule-bearing doridoidean nudibranchs were doubted due to the wide nervous system and lack of the enigmatic ‘vesicle system’ in *Helminthope*[41]. However, close relationship between both genera is supported by numerous shared morphological characters and has recently been affirmed by preliminary multi-locus sequence analyses [15,44]. Morphological characters uniting Rhodopemorpha are the wormlike, round body with no division of the body into visceral sac and headfoot, the complete loss of shell, mantle cavity (and gill) or head appendages. Internal anatomical features are 1) boomerang- or cross-shaped, verrucose spicules, 2) the reduction or loss of pharynx and radula with parallel modification of an esophageal pump, 3) pentaganglionate and euthyneurous nervous system with fused cerebral and pleural ganglia, double rhinophoral nerve roots, accessory ganglia, and paired visceral nerves, 4) monaulic genital system without allosperm receptacles or cephalic copulatory organ but with spermatophore-forming gland(s), 5) lack of heart, with protonephridial-stage kidney retained as adults, and 6) development of a caudal adhesive gland ([32], this study). However, characters 2 to 6 cannot be evaluated satisfyingly due to the lack of comparable data on the potential sister group of rhodopemorphs. Furthermore, phylogenetic analysis is hindered by meiofaunal/paedomorphic modifications found in Rhodopemorpha that involve characters commonly used to delineate Heterobranchia (Figure 7; see [17,51]), i.e. the complete loss of the shell (hyperstrophic larval shell?), mantle cavity (formation of a pallial kidney, ciliated strips, ctenidium/gill?), or due to the modification of the digestive tract (lack of a pharynx with jaws). Thus, within Heterobranchia, hypotheses on the origin of *Rhodope* and *Helminthope* from morphological and molecular data were incompatible.

Herein we reconsider newly available morphological evidence and discuss the fact that according to molecular data, rhodopemorphs are not closely related to any of the euthyneuran slugs but should instead be placed among paraphyletic ‘lower’ heterobranchs, close to the equally minute but shell-bearing, high-spired Murchisonellidae [44]. This phylogenetic position is currently counterintuitive from a morphological point of view, and similar placement was never suggested by previous authors. Not much is known about the internal anatomy of Murchisonellidae. An exception is the unusual cuticular ‘jaw’ apparatus described for murchisonellids [63,64] which implies that the radula (and pharynx?) may also be modified and largely reduced. Given these data, the reduction of pharynx and radula with parallel modification of the esophagus (elongation, vacuolization) could be a synapomorphic trait for equally minute murchisonellids and rhodopemorphs. Both also share a similar habitat, namely subtidal reef flats or rubble among seagrass [45,109]. The Caribbean *Henrya morrisoni* Bartsch, 1947 was even described as ‘infaunal’ [64].

### Heterobranch relationships revisited

Figure 7 attempts to provide an overview of current heterobranch phylogeny – which is in a state of reassembly – addressing the origin of Rhodopemorpha and mapping possible morphological characters onto a summarized version of recent molecular topologies. It includes taxa that were covered by recent molecular studies [13,14]. Some further potential ‘basal’ heterobranch taxa – e.g. the family Ringiculidae Philippi, 1853, Tjaernoeiidae Warén, 1991, ‘caenogastropod’ Cingulopsidae Fretter & Patil, 1958 (see [110]) and potentially misidentified “Pyramidellidae” – are not included due to the current lack of molecular coverage.

The origin of a possible Rhodopemorpha + Murchisonellidae clade (B in Figure 7) among Heterobranchia is still unresolved. Molecular studies [13,14] currently suggest at least four other likely monophyletic lineages at a similar phylogenetic level that are candidates for a sistergroup to the putative rhodopemorph- murchisonellid clade (see Figure 7). Those lineages are the Ectobranchia Fischer, 1884 (=Valvatoidea Gray, 1840), C) Architectonicoidea (Architectonicidae Gray, 1850 plus Mathildidae Dall, 1889) and Omalogyridae Sars, 1878, D) Aclididae Sars, 1878, and E) a monophylum of Orbitestellidae Iredale, 1917, Cimidae Warén, 1993, and the remaining Heterobranchia. The latter (F) include a monophylum of Acteonoidea + Rissoellidae Gray, 1850 (G) as sister to the Euthyneura (sensu [14]). Many of the aforementioned taxa consist mainly of small-bodied members, and detailed microanatomical studies are lacking. Therefore, published data are mostly not sufficient to evaluate homologies. For example, some ectobranch as well as other lower heterobranch species do possess an esophagus that is at least histologically similar to that of Rhodopemorpha and Murchisonellidae [51,58].

The Ectobranchia (= Valvatoidea) include planispiral, minute snails with deep-sea and limnic lineages among more conventional subtidal groups (e.g. [56,65,86]). Haszprunar et al. [51] regarded them as the most basal heterobranch offshoot retaining plesiomorphies (e.g. broad, rhipidoglossate radula in Hyalogyrinidae) and showing some unique autapomorphies such as a typical ‘ectobranch’ gill (in contrast to the general gastropod ctenidium). This topology is neither unambiguously supported nor rejected by (not yet representative) molecular results which do, however, tend to place the Ectobranchia closer to clade C. Sperm ultrastructure (see [110]) suggests that Architectonicoidea are even more basal than Ectobranchia. Also, the rhipidoglossate radula of Hyalogyrinidae is unique also among Apogastropoda and thus could alternatively be considered autapomorphic for the family rather than assuming multiple independent origins of a narrow (taenioglossate) condition in at least the ancestral caenogastropod, in non-ectobranch heterobranchs and in non-hyalogyrinid ectobranchs. Ontogenetic transitions between rhipidoglossate, grazing radulae and more narrow ones are known in vetigastropods [111], so this character may be variable also among basal heterobranchs with unknown ontogeny. We still prefer hypothesizing Ectobranchia as sister to the remaining heterobranchs, because clade A) is supported by the unique presence of ciliated strips in the mantle cavity [17]. Further but still ambiguous apomorphies of clade A) are the lack of jaws, a taenioglossate radula, and the loss of a gill. Some derived and larger-bodied taxa among A) do possess a gill (then considered to be a novel structure, [17]), broad radulae, or jaws, so alternatively these features may be convergently reduced in all/most small-bodied basal taxa. Rhodopemorphs do not share any of the aforementioned ectobranch apomorphies, and do not possess ciliary strips; the latter may be explained by the absence of a shell and mantle cavity. Exploring Murchisonellidae in microanatomical depth may also reveal their ‘jaw apparatus’ to be a reduced and narrow radula [63; BB, pers. obs.], which would fit with apomorphies of clade A). An earlier development of the mesentoblasts during ontogeny (cell 4d differentiated at the 24-cell stage, and not later) was suggested to be a shared character of “opisthobranchs and pulmonates” [51], but was also observed for *Rhodope*[35]. If not evolved convergently, we suggest this is another potential synapomorphy of clade A).

Clade C) of large-bodied Architectonicoidea (globular to planispiral Architectonicidae plus medium to high-spired Mathildidae) and minute, planispiral Omalogyridae is supported by molecular results and some possible apomorphies such as an specialized eversible proboscis besides loss of a copulatory organ (see [51,59,112]), a character that is, however, also found in clades B and D. The high-spired and minute Aclididae (D) are known to possess a ‘narrow’ radula [113], but there are no anatomical descriptions.

The monophylum E) of Orbitestellidae (small, planispiral; [80]), Cimidae (small, high-spired; [114]) and the remaining Heterobranchia is indicated by molecular results [13,14,16,115], but not yet supported (or rejected) by morphological evidence. The remaining heterobranchs (F) include a monophylum of Acteonoidea + Rissoellidae (G) as sister to Euthyneura (e.g. [13,115]). Clade F) is possibly united by the presence of giant neurons, which are, however, present in larger-bodied taxa only (see [53]). Potential apomorphies for clade G) are the bilobed head appendages (developed into a headshield in acteonoids – sometimes still with pointy corners); the shared androdiaulic condition of genital ducts of Acteonoidea and Rissoelloidea instead appears to be plesiomorphic (see [15]).

The Euthyneura (sensu [14]) comprise most of known heterobranch species diversity, and the node is robustly supported in recent multi-locus studies (for discussion see [15,116,117]). Morphological evidence for Euthyneura is less straightforward; a potential apomorphy refers to the presence of rhinophores (innervated by N3), if this is not already another synapomorphy of clade F). Rhodopemorphs do not possess any head tentacles, and the identity of the N3 (separate, or fused with N2) is ambiguous, so this feature is little informative for tracing their origin. Standard multilocus sequence marker based studies retrieve three major euthyneuran subgroups that are different from traditional morphological hypotheses, namely Nudipleura (including the speciose nudibranchs) as sister to a clade of Euopisthobranchia and Panpulmonata (e.g. [13,14]). The latter two tectipleuran clades contain rearranged lineages of traditional opisthobranchs, pulmonates, and the ‘basal heterobranch’ Glacidorbidae Ponder, 1986 and Pyramidellidae (see [13-16]). Although now contradicted by preliminary molecular results [44], older morphological studies placed Rhodopemorpha within Euthyneura based on the common possession of a euthyneurous, pentaganglionate nervous system [35,40,91]. These characters are neither unique for nor ubiquitous within Euthyneura, as is indicated by the present study. More specifically, Haszprunar and Künz [41] followed Boettger’s [118] and Odhner’s [38] proposals of including *Rhodope* within doridoidean nudibranchs (Nudipleura) due to the presence of spicules, a ‘modified’ pharynx without radula, shared reductions, and presumed ultrastructural characters. Monauly in *Rhodope* (among otherwise diaulic or triaulic nudibranchs) was explained as a consequence of paedomorphosis [41] and the occurrence of specialized mode of sperm transfer, namely hypodermal injection (see also [32]). All these characters are homoplastic in a topological framework based on molecular data (Figure 7); e.g. calcareous spicules occur convergently in rhodopemorphs, several nudipleuran subgroups [119], but also in (some) sacoglossan and acochlidian panpulmonates [22,120], pharynx reductions are common not only among nudibranchs, and paedomorphic reductions or unilateral sperm transfer are herein discussed as ‘meiofaunal syndrome’ causing similar morphology and biology in independent lineages via habitat-specific selection pressure. Therefore, the latest morphological hypothesis of rhodopemorph origin is currently neither supported by morphology nor molecular data.

Other hypotheses based on morphology placed *Rhodope* among tectipleuran Euthyneura, a clade consistently retrieved in molecular studies (e.g. [13,16]). According to recent topologies these appear to be characterized by their primarily monaulic genital ducts (see [15]), which would be consistent with a relationship to Rhodopemorpha. Diagnostic features missing in the latter such as giant neurons [17] may be reduced due to the small body size. Euopisthobranchia possess, among morphological synapomorphies, an esophageal gizzard [14,15,121]. This structure is lacking in rhodopemorphs but loss can be explained by a secondary reduction coming with small body size, as a gizzard is also missing e.g. in the meiofaunal philinoglossid cephalaspideans [21,26]. In fact, morphology-based cladistic studies [121], see also [122] recovered *Rhodope* clustering with meiofaunal Cephalaspidea (Euopisthobranchia) and panpulmonate Acochlidia. This particular grouping is polyphyletic according to molecular results (see [14]), suggesting that it is a result of homoplasies (‘meiofaunal syndrome’) overriding other morphological characters [15,120]. Other authors assumed rhodopemorph affiliations to panpulmonate Gymnomorpha, i.e. Onchidiidae, based on *Rhodope* possessing a putative mantle cavity – herein shown to be erroneous – and a highly concentrated nervous system [19,35,39,123]. This placement was later doubted due to the lack of the diagnostic pulmonate neurosecretory procerebrum in rhodopemorphs [29,40]. However, as outlined above, the double-rooted rhinophoral ganglion of *Rhodope* could still prove to be homologous to the double-rooted procerebrum, and thus the double roots could be interpreted as a synapomorphy of (many) panpulmonates and rhodopemorphs. This interpretation is, however, in conflict with general morphology and structural differences weakening homology probability; in rhodopemorphs there are no ‘globineurons’ as typical for the pulmonate procerebrum [40,87,88]. Molecular results (Figure 7) indicate that a double-rooted rhinophoral nerve has evolved independently in rhodopemorphs and panpulmonates and thus constitute potential apomorphies of the respective groups.

## Conclusions

Microanatomical exploration of rhodopemorphs provides strong evidence that the aberrant morphology of members refers to features (complex nervous system, presence of spicules, special reproductive strategies, adhesive glands) and regressive processes we account to a taxonomically widespread ‘meiofaunal syndrome’. We interpret *Helminthope*, the most worm-like free-living gastropod, to be a progenetic sister of *Rhodope*, i.e. referring to an over-elongate and premature larval stage. We explore the diverse and largely incompatible previous morphology-based hypotheses on the origin of rhodopemorphs among heterobranch gastropods. Any earlier proposed relationships to euthyneuran opisthobranchs are not supported in the light of currently available microanatomical data, and are contradicted by (still preliminary) molecular evidence. Should future molecular studies corroborate placement of Rhodopemorpha among ‘lower heterobranch’ taxa, then more knowledge is needed on the minute, shelled basal heterobranch groups for better resolution and support for future phylogenies. 3D reconstruction has been demonstrated to be suited for anatomical examination of small-bodied taxa, and should be equally useful for studies on still barely known heterobranch groups such as Murchisonellidae, Aclididae, Cimidae, or the legions of snails that are currently pooled into vetigastropod or caenogastropod taxa just for their small size and shell features. Especially murchisonellids need anatomical study to test for possible anatomical synapomorphies with rhodopemorphs.

Because murchisonellid genera have been shown to exist as ‘living fossils’ since the Triassic [45], the putative murchisonellid-rhodopid split is potentially almost as old. The basal phylogenetic position of rhodopemorphs therefore makes them a candidate for the oldest lineage of meiofaunal slugs, and also for one of the oldest living slug lineages at all. Rhodopemorphs represent a fascinating, highly modified gastropod taxon among the otherwise typical snail-like lower heterobranchs, and give valuable insight into the enormous evolutionary potential of that much larger group.

## Material and methods

About 20 specimens of *Helminthope psammobionta* Salvini-Plawen, 1991 were extracted from bulk samples of coarse subtidal sand taken from 2–4 meters depth at Police bay, Bermuda (close to the type locality), during October 1999. Specimens were anesthetized using isotonic magnesium chloride solution mixed with seawater, then fixed in 4% glutaraldehyde. All vouchers are stored at the Bavarian State Collection of Zoology, Munich (ZSM).

Several glutaraldehyde-fixed specimens were postfixed with 1% osmium tetroxide buffered with 0.2 M cacodylate / 0.3 M sodium chloride, then dehydrated in a graded acetone series and embedded in Spurr’s epoxy resin.

3D reconstruction was done following largely the protocol described by Ruthensteiner [124]. Series of semithin histological sections (1 μm) were obtained using a diamond knife (Diatome HistoJumbo, Biel, Switzerland) and stained with methylene blue/azure II stain [125]. Photographs were taken of each section using a ProgRes C3 digital camera (Jenoptik, Jena, Germany) mounted on a Leica DMB-RBE microscope (Leica Microsystems, Wetzlar, Germany). Digital images were imported into Amira 5.2 software (Visage Imaging, Berlin, Germany) as greyscale .*tif*-files with a resolution of 1600 × 1200 dpi. Images were aligned semi-automatically and organ systems labeled manually on the screen. From these labels, rendered 3D models were created of an entire, moderately contracted 1.5 mm specimen (ZSM Mol-19992019/2; 613 photos used; see Figure 1), the kidney of this specimen (61 photos; Figure 1B) and of the anterior body containing the central nervous system (CNS) of another 3 mm specimen (ZSM Mol-19992020/2; 358 photos; see Figure 3). Additional aligned image stacks of approximately 100 images with higher resolution and color were used to analyze very small features present in the aforementioned specimens. Histological features were furthermore compared with section series of two further specimens (ZSM Mol 20120177 and 20120178).

Interactive models of the 3D reconstructions were prepared following the protocol of Ruthensteiner and Heß [126], and are accessible as two clickable Additional files 1 and 2.

## Competing interests

The authors declare that they have no competing interests.

## Authors’ contributions

BB carried out the morphological analysis and drafted the manuscript. GH supplied materials and unpublished information and discussed results. MS conceived and supervised the study and helped writing the paper. All authors read and approved the final manuscript.

## Supplementary Material

Additional file 1: Figure S1 3D reconstruction of *H. psammobionta* (ZSM Mol-19992019/2) showing organization of major organ systems, anterior to the right. **A**: Right view of complete, moderately contracted specimen. **B**: Kidney of same specimen, dorsal view. **C**: Reproductive system. Scale bars: A, 100 μm; B, 25 μm; C, 50 μm. Abbreviations: ag, accessory ganglia; agl, caudal adhesive gland; am, ampulla; an, anus; apg, anterior pedal glands; bb, buccal bulb; cpg, cerebropleural ganglion; dg, digestive gland; ey, eye; fg1-5, female glands (proximal to distal); fz, presumed filter zone; gd, (undifferentiated) gonoduct; go, gonad; gp, genital pore; it, intestine; kd, kidney; mo, mouth opening; np, nephropore; oc, oocytes; pg, pedal ganglia; sgl, salivary gland; tg, ‘terminal’ gland; vg, visceral ganglion; vn, visceral nerves. Click to activate interactive 3D model (requires Adobe Reader 7.0 or higher). Use mouse to rotate model, shift model (hold ctrl) or zoom (use mouse wheel). Switch between prefabricated views or select components in the model tree and change visualization (e.g. transparency, lighting, render modes, or crop). Click here for file

Additional file 2: Figure S3 3D reconstruction of the anterior end of an extended *H. psammobionta* (ZSM Mol-19992020/2) showing details of the central nervous system (cns), anterior to the right. **A**: Dorsal view of cns. Digestive system transparent, pedal nerves omitted. **A’**: The reconstructed specimen prior to sectioning, box marks region shown in this figure. **B**: Ventral view of ganglia, digestive system, and retractor muscle. Nerves largely omitted. **C**: Dorsal right view of anterior cns and details of the cerebral innervation. Pedal nerves transparent. Scale bars: 100 μm. Abbreviations: see main document Figure 3. Click to activate interactive 3D model (requires Adobe Reader 7.0 or higher). Use mouse to rotate model, shift model (hold ctrl) or zoom (use mouse wheel). Switch between prefabricated views or select components in the model tree and change visualization (e.g. transparency, lighting, render modes, or crop). Click here for file
