# Supplementary material for: At the limits of a successful body plan – 3D microanatomy, histology and evolution of Helminthope (Mollusca: Heterobranchia: Rhodopemorpha), the most worm-like gastropod
Source: Front Zool. 2013 Jun 28;10:37. doi: 10.1186/1742-9994-10-37 (PMC3704743; doi:10.1186/1742-9994-10-37)
Supplement: Additional file 1: Figure S1 — 3D reconstruction of H. psammobionta (ZSM Mol-19992019/2) showing organization of major organ systems, anterior to the right. A: Right view of complete, moderately contracted specimen. B: Kidney of same specimen, dorsal view. C: Reproductive system. Scale bars: A, 100 μm; B, 25 μm; C, 50 μm. Abbreviations: ag, accessory ganglia; agl, caudal adhesive gland; am, ampulla; an, anus; apg, anterior pedal glands; bb, buccal bulb; cpg, cerebropleural ganglion; dg, digestive gland; ey, eye; fg1-5, female glands (proximal to distal); fz, presumed filter zone; gd, (undifferentiated) gonoduct; go, gonad; gp, genital pore; it, intestine; kd, kidney; mo, mouth opening; np, nephropore; oc, oocytes; pg, pedal ganglia; sgl, salivary gland; tg, ‘terminal’ gland; vg, visceral ganglion; vn, visceral nerves. Click to activate interactive 3D model (requires Adobe Reader 7.0 or higher). Use mouse to rotate model, shift model (hold ctrl) or zoom (use mouse wheel). Switch between prefabricated views or select components in the model tree and change visualization (e.g. transparency, lighting, render modes, or crop). [file 1742-9994-10-37-S1.pdf]

Supplementary file 1

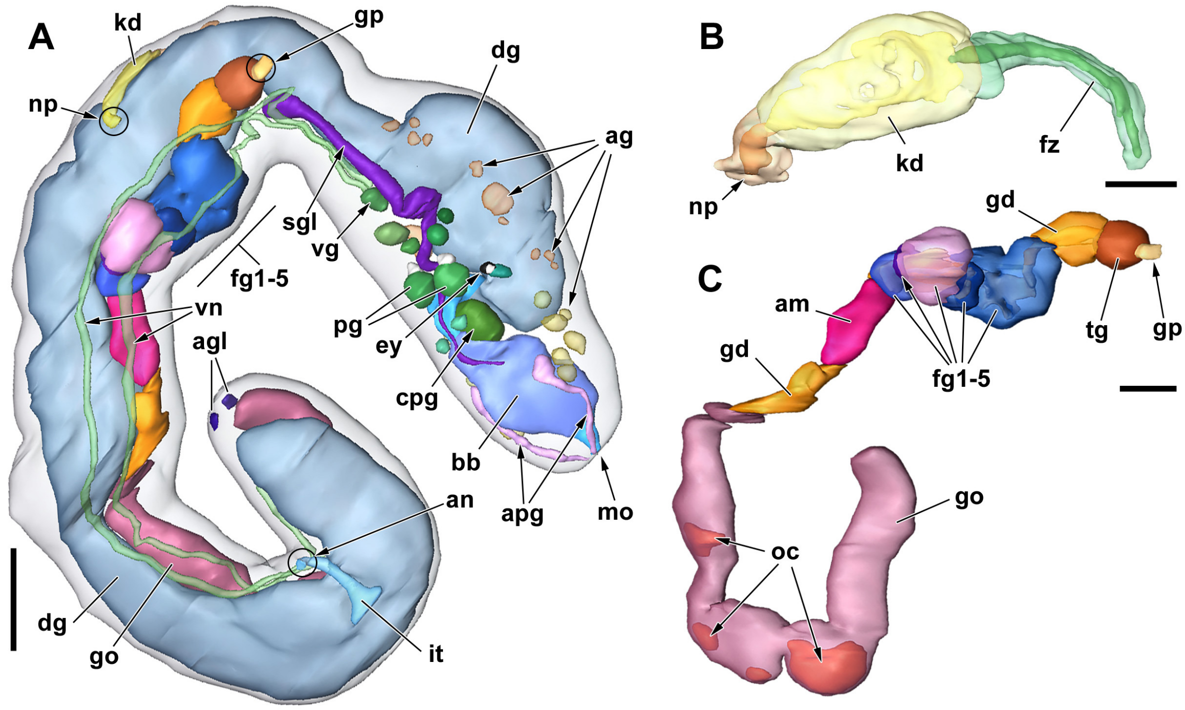

Interactive Fig. 1. 3D reconstruction of *H. psammobionta* (ZSM Mol-19992019/2) showing organization of major organ systems, anterior to the right.

A: Right view of complete, moderately contracted specimen. **B:** Kidney of same specimen, dorsal view. **C:** Reproductive system. Scale bars: A, 100 µm; B, 25 µm; C, 50 µm.

Abbreviations: ag, accessory ganglia; agl, caudal adhesive gland; am, ampulla; an, anus; apg, anterior pedal glands; bb, buccal bulb; cpg, cerebropleural ganglion; dg, digestive gland; ey, eye; fg1-5, female glands (proximal to distal); fz, presumed filter zone; gd, (undifferentiated) gonoduct; go, gonad; gp, genital pore; it, intestine; kd, kidney; mo, mouth opening; np, nephropore; oc, oocytes; pg, pedal ganglia; sgl, salivary gland; tg, 'terminal' gland; vg, visceral ganglion; vn, visceral nerves.

Click to activate **interactive 3D model** (requires Adobe Reader 7.0 or higher). Use mouse to rotate model, shift model (hold ctrl) or zoom (use mouse wheel). Switch between prefabricated views, or select components in the model tree and change visualization (e.g. transparency, lighting, render modes, or crop).
