# Supplementary material for: At the limits of a successful body plan – 3D microanatomy, histology and evolution of Helminthope (Mollusca: Heterobranchia: Rhodopemorpha), the most worm-like gastropod
Source: Front Zool. 2013 Jun 28;10:37. doi: 10.1186/1742-9994-10-37 (PMC3704743; doi:10.1186/1742-9994-10-37)
Supplement: Additional file 2: Figure S3 — 3D reconstruction of the anterior end of an extended H. psammobionta (ZSM Mol-19992020/2) showing details of the central nervous system (cns), anterior to the right. A: Dorsal view of cns. Digestive system transparent, pedal nerves omitted. A’: The reconstructed specimen prior to sectioning, box marks region shown in this figure. B: Ventral view of ganglia, digestive system, and retractor muscle. Nerves largely omitted. C: Dorsal right view of anterior cns and details of the cerebral innervation. Pedal nerves transparent. Scale bars: 100 μm. Abbreviations: see main document Figure 3. Click to activate interactive 3D model (requires Adobe Reader 7.0 or higher). Use mouse to rotate model, shift model (hold ctrl) or zoom (use mouse wheel). Switch between prefabricated views or select components in the model tree and change visualization (e.g. transparency, lighting, render modes, or crop). [file 1742-9994-10-37-S2.pdf]

Supplementary file 2

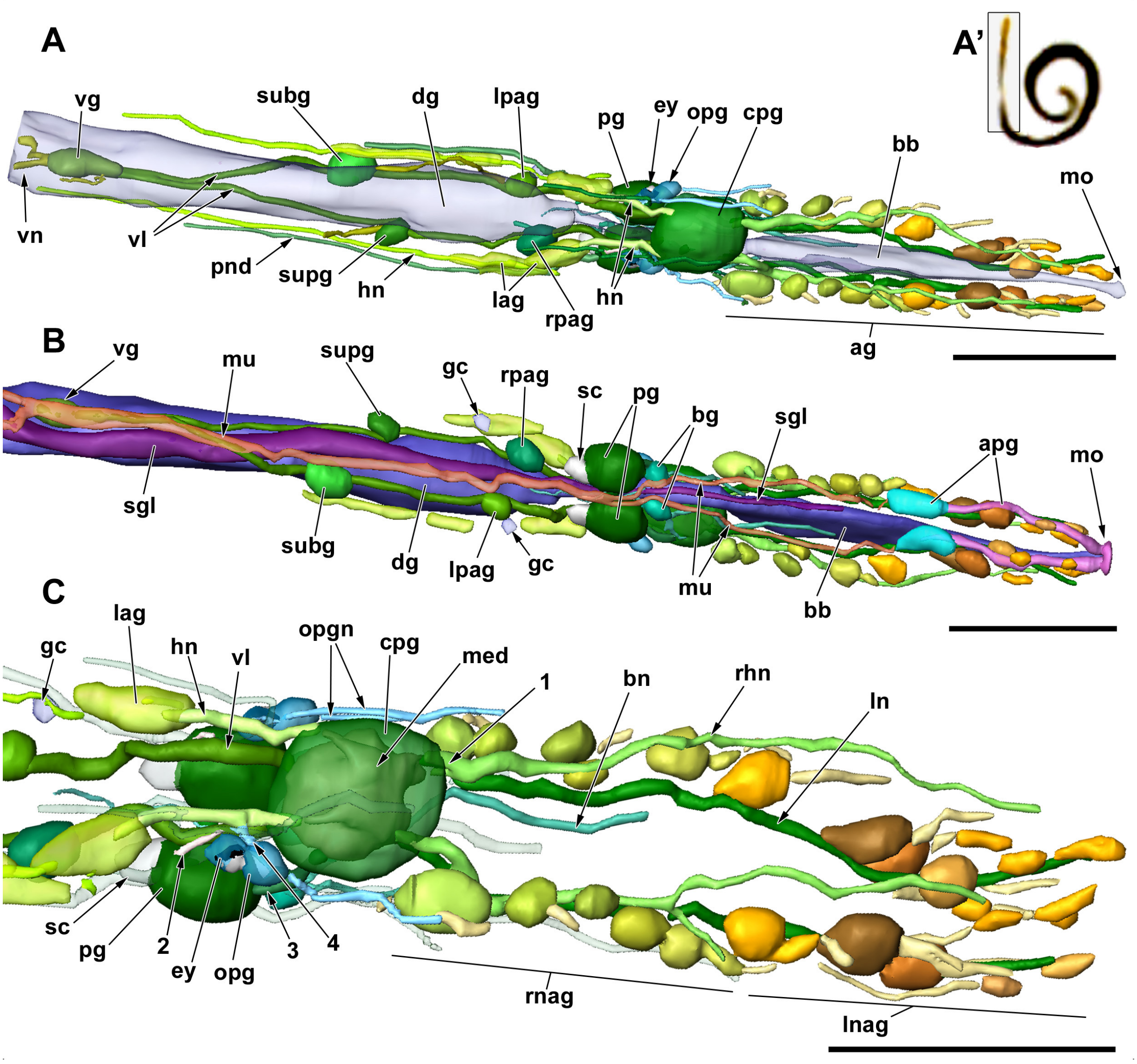

Interactive Fig. 4. 3D reconstruction of the anterior end of an extended *H. psammobionta* (ZSM Mol-19992020/2) showing details of the central nervous system (cns), anterior to the right.

A: Dorsal view of cns. Digestive system transparent, pedal nerves omitted. **A’:** The reconstructed specimen prior to sectioning, box marks region shown in this figure. **B:** Ventral view of ganglia, digestive system, and retractor muscle. Nerves largely omitted. **C:** Dorsal right view of anterior cns and details of the cerebral innervation. Pedal nerves transparent. Scale bars: 100 µm. Abbreviations: see main document Fig. 4. Click to activate **interactive 3D model** (requires Adobe Reader 7.0 or higher). Use mouse to rotate model, shift model (hold ctrl) or zoom (use mouse wheel). Switch between prefabricated views, or select components in the model tree and change visualization (e.g. transparency, lighting, render modes, or crop).
